# Transplantation of active nucleus pulposus cells with a keep-charging hydrogel microsphere system to rescue intervertebral disc degeneration

**DOI:** 10.1186/s12951-023-02226-1

**Published:** 2023-11-28

**Authors:** Yingchuang Tang, Kai Zhang, Hongyou Zhou, Chenchen Zhang, Zixiang Liu, Hao Chen, Hanwen Li, Kangwu Chen

**Affiliations:** 1 Department of Orthopedic, First Affiliated Hospital of Soochow University, Suzhou, People’s Republic of China; 2 Department of Radiology, Second Affiliated Hospital of Soochow University, Suzhou, People’s Republic of China; 3 Department of Orthopedics, Affiliated Hospital of Yangzhou University, Yangzhou, People’s Republic of China; 4 Institute of Translational Medicine, Medical College, Yangzhou University, Yangzhou, People’s Republic of China

**Keywords:** Intervertebral disc degeneration, Nucleus pulposus cells transplantation, Hydrogel microspheres, Magnesium ion

## Abstract

**Background:**

Cell transplantation has been demonstrated as a promising approach in tissue regeneration. However, the reactive oxygen species (ROS) accumulation and inflammation condition establish a harsh microenvironment in degenerated tissue, which makes the transplanted cells difficult to survive.

**Methods:**

In this study, we constructed a keep-charging hydrogel microsphere system to enable cells actively proliferate and function in the degenerated intervertebral disc. Specifically, we combined Mg^2+^ to histidine-functionalized hyaluronic acid (HA-His-Mg^2+^) through coordination reaction, which was further intercrossed with GelMA to construct a double-network hydrogel microsphere (GelMA/HA-His-Mg^2+^, GHHM) with microfluidic methods. In vitro, the GHHM loaded with nucleus pulposus cells (GHHM@NPCs) was further tested for its ability to promote NPCs proliferation and anti-inflammatory properties. In vivo, the ability of GHHM@NPCs to promote regeneration of NP tissue and rescue intervertebral disc degeneration (IVDD) was evaluated by the rat intervertebral disc acupuncture model.

**Results:**

The GHHM significantly enhanced NPCs adhesion and proliferation, providing an ideal platform for the NPCs to grow on. The loaded NPCs were kept active in the degenerative intervertebral disc microenvironment as charged by the Mg^2+^ in GHHM microspheres to effectively support the loaded NPCs to reply against the ROS-induced inflammation and senescence. Moreover, we observed that GHHM@NPCs effectively alleviated nucleus pulposus degeneration and promoted its regeneration in the rat IVDD model.

**Conclusion:**

In conclusion, we constructed a keep charging system with a double-network hydrogel microsphere as a framework and Mg^2+^ as a cell activity enhancer, which effectively maintains NPCs active to fight against the harsh microenvironment in the degenerative intervertebral disc. The GHHM@NPCs system provides a promising approach for IVDD management.

**Graphical Abstract:**

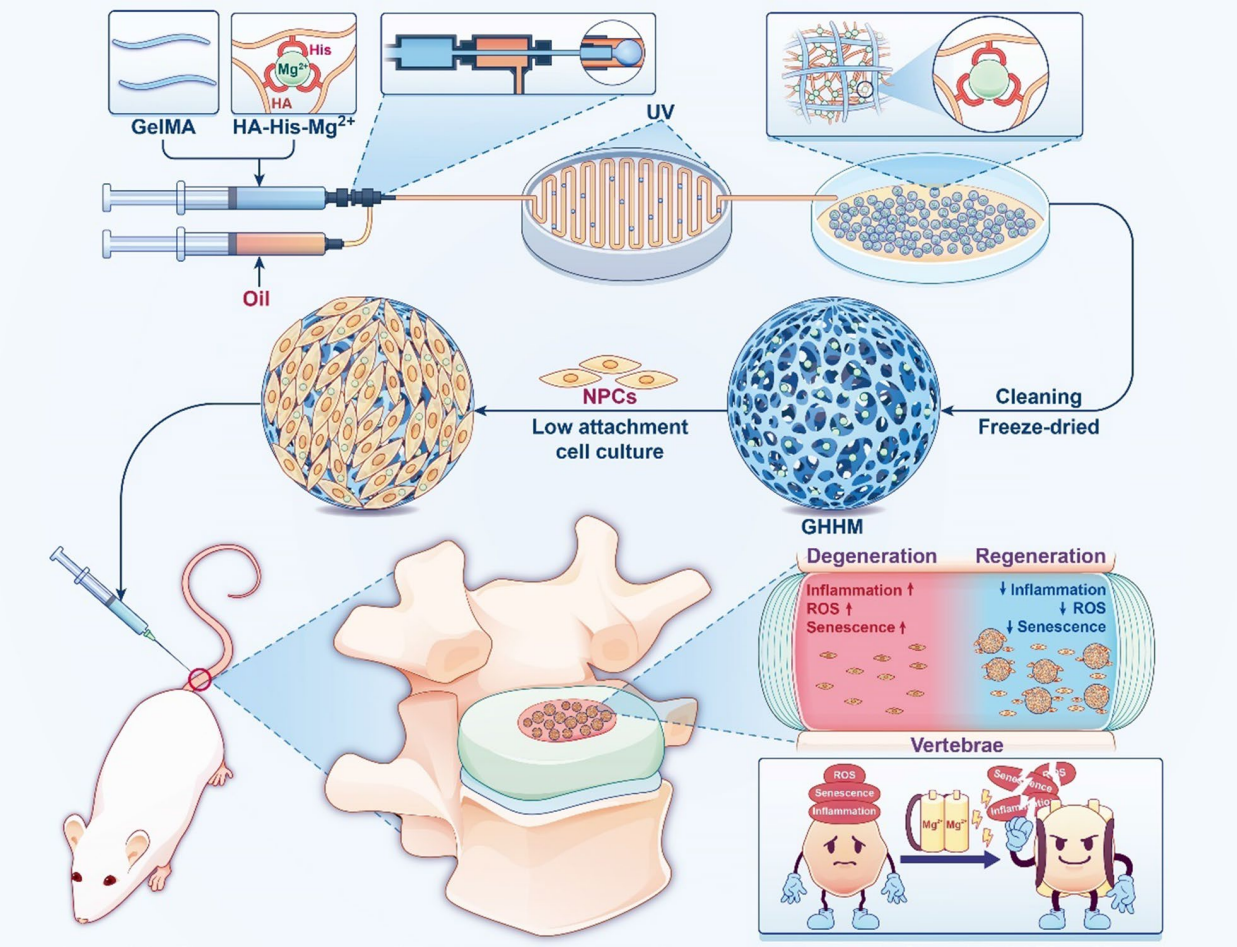

**Supplementary Information:**

The online version contains supplementary material available at 10.1186/s12951-023-02226-1.

## Introduction

Intervertebral disc degeneration (IVDD) is a worldwide disorder that seriously interferes human's life quality [1–3]. Conservative treatment is always working unsatisfactory [3, 4], while surgical treatment is traumatic to patients [5, 6]. Therefore, researchers are striving to regenerate disc or prevent the disc from collapsing through biological approaches [7–9]. The nucleus pulposus tissue is located within a relatively closed region where no blood vessels or nerves are distributed. With the characteristics, the degenerative nucleus pulposus tissue is not conducive to regenerate [10]. Meanwhile, during the process of IVDD, a cytokine storm [11] and excess reactive oxidation from mitochondrial failure [8, 9, 12] in the intervertebral disc (IVD) prevent researchers to establish the effective way for its regeneration. Inhibition of the inflammation locally may slow the IVDD progress but does not appear to recover the injured tissues [13, 14]. Li et al. constructed an oxygen metabolism-balanced engineered hydrogel microsphere loaded with black phosphorus quantum dots, which attenuates the inflammatory cascade and thus delays disc degeneration [15]. Moreover, a high-strength hydrogel based on zinc-oxidized sodium alginate-gelatin as a multifunctional nucleic acid delivery platform was also designed for the treatment of IVDD [16]. These biomaterials may reduce the extracellular matrix (ECM) anabolic/catabolic imbalance by inhibiting the inflammatory storm, but do not hold the ability to regenerate the nucleus pulposus tissue [17].

Cell transplantation has been proved to be a practical method for tissue regeneration. Sakai Daisuke et al. had detailed the effectiveness of cell transplantation for IVDD [18, 19]. Hu et al. [20] developed a gene-cell combination hydrogel system to treat IVDD. However, the confinement and the immunity make it difficult for the transplanted cells to proliferate [9]. Peng et al. [21] analyzed the current difficulties of cell transplantation for IVDD, showing the avascular, hypoglycemic, hypoxic tension, low pH and nutrient-deficient disc environment are some of the unfavorable factors that must be overcome for the survival of transplanted cells after transplantation [22, 23]. Therefore, it is necessary to construct a cell transplantation system that could enhance cellular activity in a long term that couple with the degenerative microenvironment.

Several studies reported small molecules are proteopeptides that tend to fail in degenerate microenvironments because of acidic inflammation, whereas metal ions are mostly unaffected [24, 25]. Mineral ions (e.g., calcium, copper, zinc, and magnesium) have been shown in recent researches to play vital roles when combine with biological materials, offering outstanding anti-bacterial [26, 27], anti-oxidant [28], and osteogenic qualities [29]. Magnesium ion (Mg^2+^) is an essential trace element that plays an important role in human metabolism and in cellular aging [30–32]. Mg^2+^ is required for DNA replication and is directly engaged in cell fate determination [30, 31]. Moreover, Mg^2+^ deficiency causes increased inflammation and oxidative stress, while adding Mg^2+^ in vitro or in vivo reduces inflammatory response and phagocytic activation [33, 34]. As a carrier, the injectability and biocompatibility of hydrogels are attractive, so we aim to combine it with Mg^2+^ to design a cell delivery material, which is capable to maintain the activity of the transplanted NPCs to resist the harsh microenvironment and promote nucleus pulposus regeneration. However, simply physical blend would let the Mg^2+^ to release quickly. Hence, we used the coordination reaction of metal coordinate bonds to create histidine-functionalized hyaluronic acid (HA-His-Mg^2+^). Considering the rapid degradation of single HA hydrogel, a double-network hydrogel microsphere was designed to increase the stability. Gelatin methacryloyl (GelMA) is commonly used injectable hydrogel materials with a three-dimensional structure suitable for cell growth and differentiation, ideal biocompatibility and cellular reactivity [35, 36]. Then, the double-network hydrogel microsphere system (GelMA/HA-His-Mg^2+^, GHHM) was constructed with GelMA by using a microfluidic method.

In this study, we used Magnesium ion as a cell activity enhancer. After full load of NPCs (GHHM@NPCs), the GHHM continuously charged to keep the loaded NPCs active. The GHHM@NPCs are integrated and capable to regulate the ROS-induced inflammation, rescuing the loaded and surrounded NPCs from senescence. Then, the GHHM@NPCs continuously proliferate to regenerate nucleus pulposus tissue. The constructed keep-charging hydrogel microsphere system presents strong potential for IVDD regeneration and is expected to translate for clinical practice.

## Results

### Construction and characterization of the GHHM microspheres

**Fig. 1:**
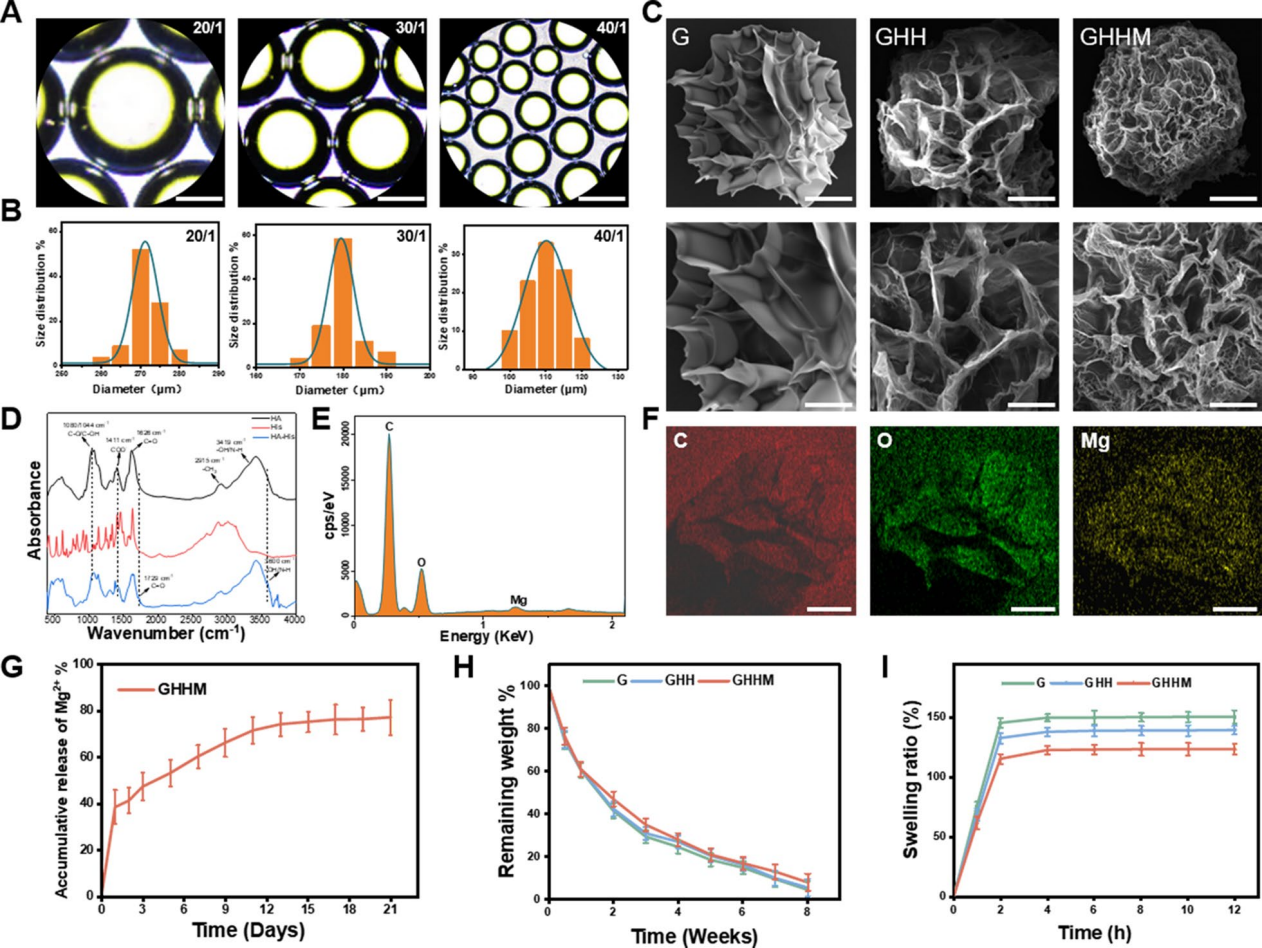
Construction and characterization of GHHM microspheres. **A** Microsphere observation in the light field, scale bar = 100 μm. **B** Distribution of particle size at different flow rates ratios. **C** SEM images of G, GHH and GHHM microspheres, scale bar above = 50 μm, below = 25 μm. **D** FTIR analysis of HA-His. **E** EDS spectrum of GHHM microspheres. **F** SEM Mapping of GHHM microspheres, scale bar = 50 μm. **G** Release curve of Mg^2+^. **H** Degradation curves of G, GHH and GHHM microspheres in PBS. **I** Dissolution curves of G, GHH and GHHM microspheres

Firstly, Mg^2+^ was successfully encapsulated into histidine-functionalized hyaluronic acid by the coordination reaction of metal ion ligands (HA-His-Mg^2+^). Then, it was combined with GelMA to construct a double-network hydrogel microsphere (GelMA/HA-His-Mg^2+^, GHHM), which was produced by a microfluidic system combined with UV-light for cross-linking. At the meantime, GelMA (G), GelMA/HA-His (GHH) and GelMA/HA-His-Mg^2+^ (GHHM) hydrogel microspheres were constructed as different groups for the following assays. The microspheres appeared with different diameters as caused by the various ratios between the oil and water phases. It was observed that the hydrogel microspheres with different sizes all exhibited excellent dispersion and spherical integrity. However, the increased ratio made the diameter of the microsphere smaller and less uniform (Fig. 1A). Under the flow rate ratios of the oil versus water phases with 20:1, 30:1 and 40:1, the diameters of the microsphere were 270 ± 26 μm, 180 ± 15 μm and 110 ± 32 μm, respectively (Fig. 1B). Considering the requirements of homogeneity and injectability, the hydrogel microspheres with a flow rate ratio of 30:1 was finally selected (Additional file 1: Figure S1). SEM images showed GHHM preserved more sparse porous structures following lyophilization than GelMA microspheres (Fig. 1C). Fourier transform infrared spectroscopy (FTIR) revealed the relative intensities of the absorption peaks of the samples' C–OH and COO- structures at 1044 cm^−1^ and 1411 cm^−1^ were significantly weakened, indicating the structures such as the hydroxyl and carbonyl groups of hyaluronic acid are key cross-linking sites for histidine N–H, and hyaluronic acid was successfully cross-linked with histidine (Fig. 1D). Both energy dispersive spectroscopy (EDS) and SEM Mapping showed homogeneous distribution of Mg^2+^ elements in the GHHM (Fig. 1E, F). The GHHM was found to continuously release Mg^2+^ for more than 21 days (Fig. 1G). By measuring the amount of Mg^2+^ released from GHHM, it reflected that the degradation of all the three kinds of microspheres lasted for more than 8 weeks, with which the curves of the three kinds of microspheres were similar (Fig. 1H). Moreover, GHHM has the lowest swelling rate, indicating its ability in maintaining the original shape well along the degradation process (Fig. 1I). Based on the above results, we confirmed successful synthesize of the GHHM microspheres, which show suitable properties in solubilization and degradation, and also has the ability to release magnesium ions in a steady pace. The GHHM microsphere laid down a foundation for the following experiments.

### The biocompatibility and cell adhesion property of the GHHM microspheres

**Fig. 2:**
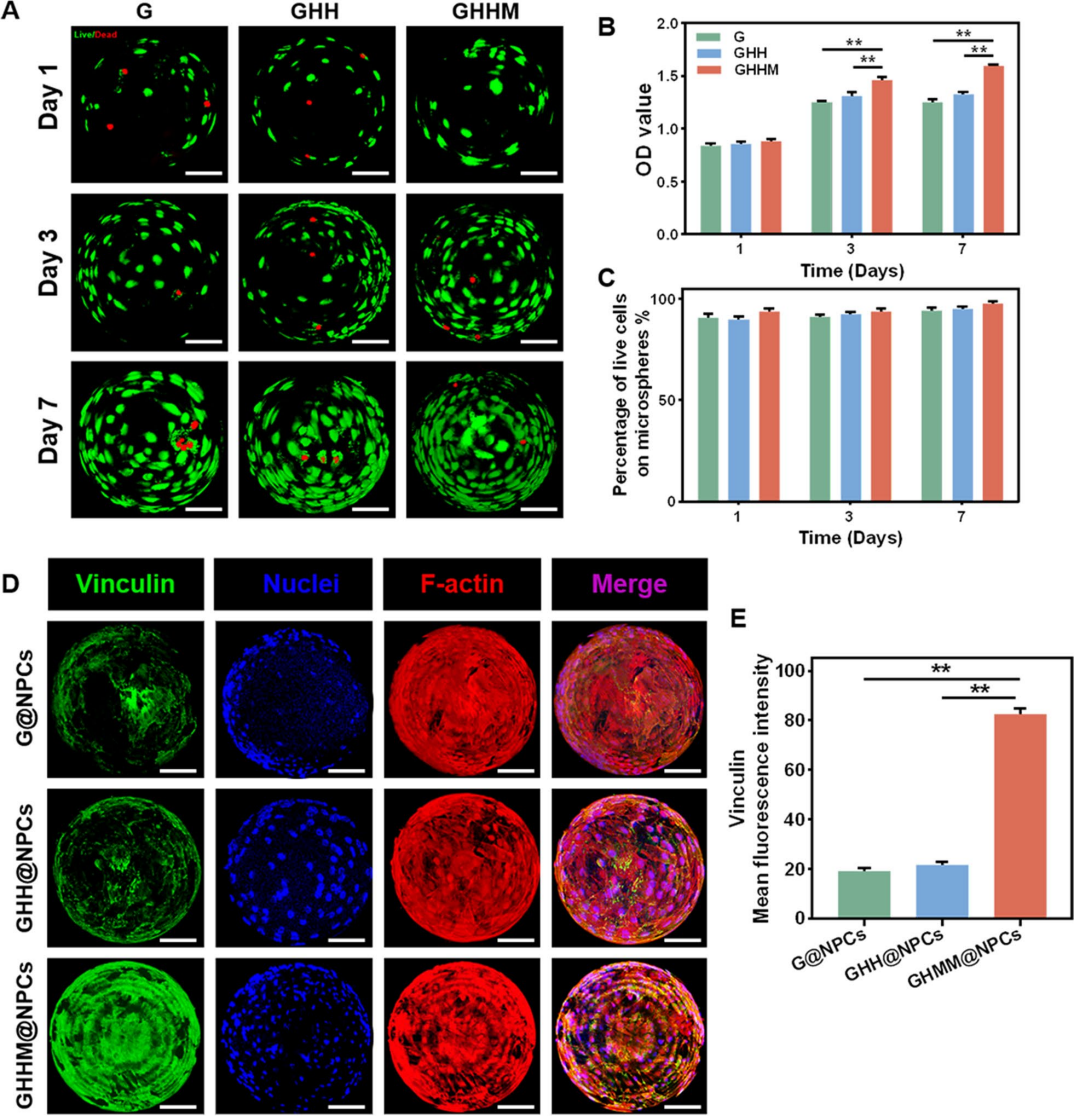
The biocompatibility and cell adhesion property of the GHHM microspheres in vitro. **A** Live/Dead staining of cells on G, GHH and GHHM microspheres, scale bar = 50 μm. **B** CCK-8 assay of different microspheres (compared with G group). **C** Percentage of live cells on G, GHH and GHHM microspheres. **D** Quantitative analysis of mean fluorescence intensity of Vinculin, scale bar = 50 μm. **E** Quantitative analysis of Vinculin. **p < 0.01

To test the biocompatibility of the GHHM microspheres, Live/Dead staining and CCK-8 assay were performed. The cytotoxicity of Mg^2+^ was first assessed by Live/Dead staining of NPCs under different concentrations of Mg^2+^ treatment. After 7 days culture, the cell density in the 100 mM Mg^2+^ group was significantly lower than that in the lower concentration groups, while the NPCs in the 200 mM group were almost dead (Additional file 1: Figure S2A, B). Furthermore, the proliferation rate of the NPCs after 1-, 3- and 7-days culture in different concentrations of Mg^2+^ was tested by CCK-8 kit. The results showed that 25 mM and 50 mM groups were less toxic to the NPCs, which even present mild enhancing effect in proliferation. When the concentration of Mg^2+^ increased to 100 mM or 200 mM, the proliferation ability of the NPCs was significantly inhibited (Additional file 1: Figure S2C). Therefore, 50 mM Mg^2+^ was chosen as an appropriate concentration for GHHM microspheres construction. Live/Dead staining and CCK-8 assays on days 1, 3 and 7 showed the NPCs on all three kinds of microspheres presented high survival rates, demonstrating ideal proliferation potential (Fig. 2A, B and C). Next, the cell adhesion property of the loaded NPCs on microspheres was evaluated by immunofluorescence staining (IF) of Vinculin. The results showed that NPCs on the GHHM microspheres expressed the most abundant fluorescence signal, representing satisfactory cell adhesion capacity (Fig. 2D, E). Overall, the above results showed that the GHHM microspheres are biocompatible, providing a viable platform for NPCs transplantation.

### The GHHM microspheres keep the loaded NPCs (L-NPCs) active to fight against the degenerative microenvironment in IVDD

**Fig. 3:**
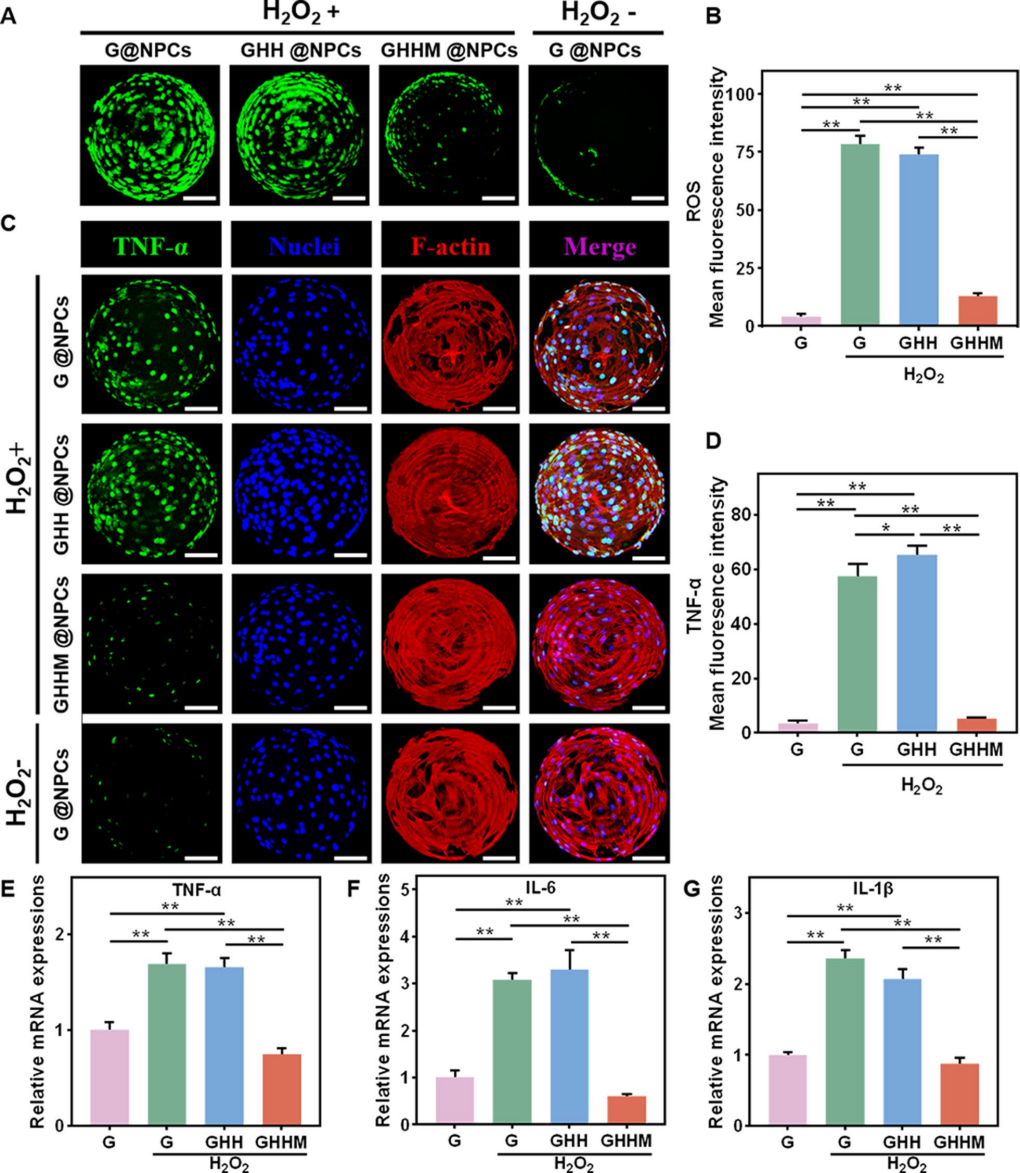
Effects against ROS-induced inflammation of GHHM microspheres in vitro. **A** ROS analysis of L-NPCs on G/GHH/GHHM microspheres after 200 μM H_2_O_2_ treatment, scale bar = 50 μm. **B** Quantitative analysis of mean fluorescence intensity of ROS. **C** Immunofluorescence analysis of TNF-α, scale bar = 50 μm. **D** Quantitative analysis of mean fluorescence intensity of TNF-α. **E**–**G** RT-qPCR analysis of the relative mRNA expression of the inflammatory-related genes after different treatment. *p < 0.05, **p < 0.01

To imitate the degenerative microenvironment experienced by NPCs during IVDD, H_2_O_2_ was employed to establish a highly inflammatory and ROS microenvironment in vitro [37], Then, the Live/Dead staining and CCK-8 assay were carried out to evaluate the cell viability. After 72 h culture of NPCs in the medium with different concentrations of H_2_O_2_, it showed that the cell viability decreased to 70.7 ± 3.7% and 53.77 ± 4.1% at 200 μM and 400 μM, respectively (Additional file 1: Figure S3A, B), of which, the concentration of 200 μM was chosen for the following tests. The ROS and inflammation status of the L-NPCs on the GHHM microspheres were evaluated after 3 days of treatment with H_2_O_2_ (200 μM). ROS kit revealed the ROS level of the L-NPCs was increased both in the G and GHH groups after H_2_O_2_ treatment, while it was significantly decreased back to the baseline in the GHHM group (Fig. 3A, B). In vitro, TNF-α staining was performed to evaluate the anti-inflammation ability of the microspheres. Compare with the G and GHH groups, TNF-α expression in the L-NPCs was significantly decreased in the GHHM group, which was similar as that in the ROS evaluation (Fig. 3C, D). In addition, RT-qPCR showed the expression of inflammation-related genes, including IL-6, IL-1β and TNF-α, in the L-NPCs was also down-regulated in the GHHM group (Fig. 3E–G).

**Fig. 4:**
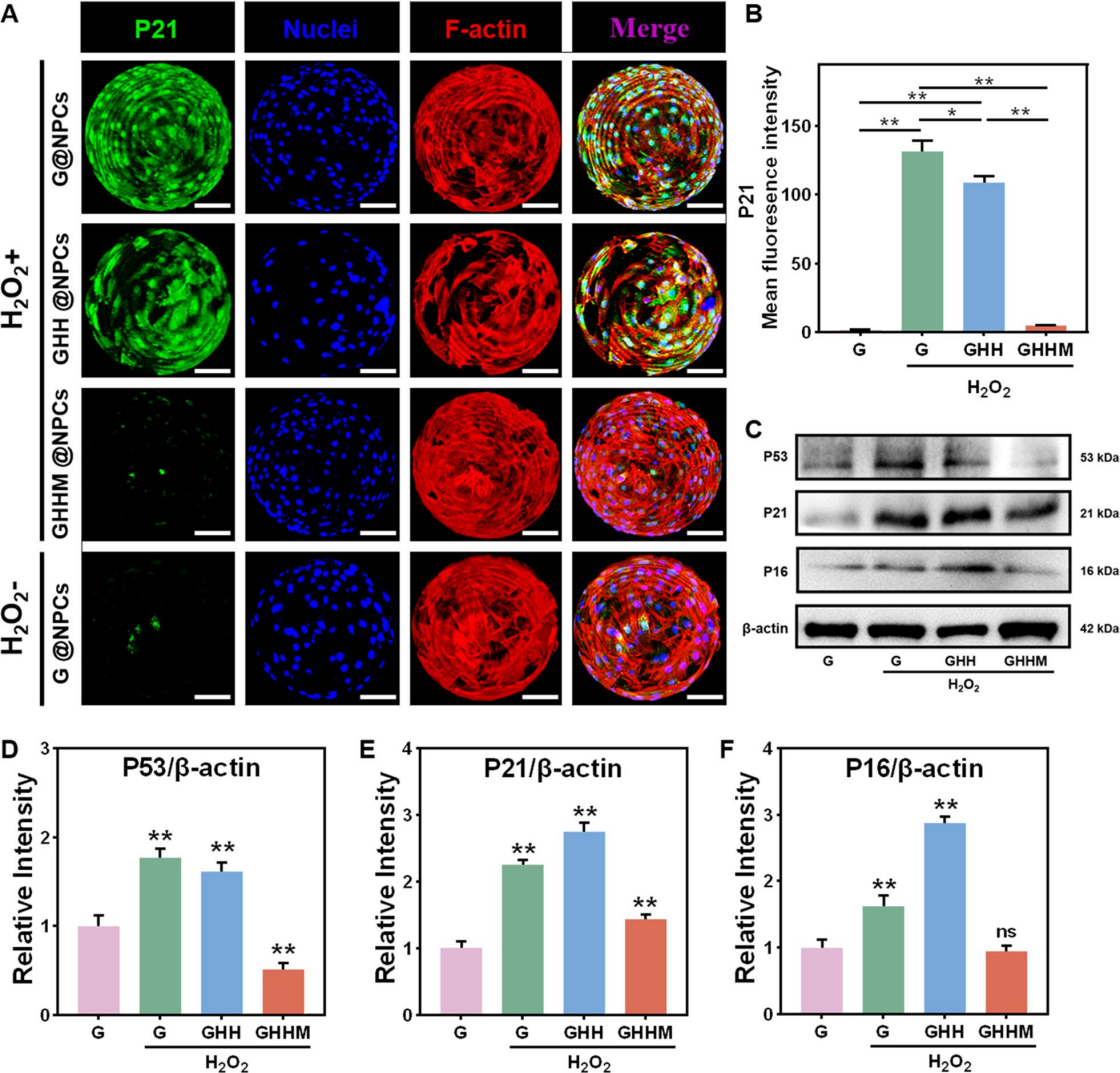
The ability of GHHM microspheres to retard the L-NPCs senescence in vitro. **A** Immunofluorescence analysis of P21, scale bar = 50 μm. **B** Quantitative analysis of mean fluorescence intensity of P21. **C** Western blot of the senescence-related proteins in L-NPCs treated with H_2_O_2_. **D**–**F** Relative protein quantification of grey scale value for senescence-related proteins (ratio to β-actin), compared with the control group. *p < 0.05, **p < 0.01, *ns* no significant difference

As cellular senescence is the major result of ROS, therefore, the anti-senescence ability of GHHM was further evaluated in vitro. Data showed that the P16 and P21 staining of the L-NPCs on the GHHM was less expressed than the other groups, indicating the GHHM has an ideal ability to resist cellular senescence in the degenerative microenvironment (Fig. 4A, B and Additional file 1: Figure S4). Meanwhile, the Western Blot (WB) results also revealed similar results as reflected by the IF staining (Fig. 4C–F). The above results confirmed that the GHHM enhanced the viability of L-NPCs to scavenge ROS and against ROS-induced inflammation or cellular senescence.

### The GHHM microspheres maintain the L-NPCs with original bio-characteristics

**Fig. 5:**
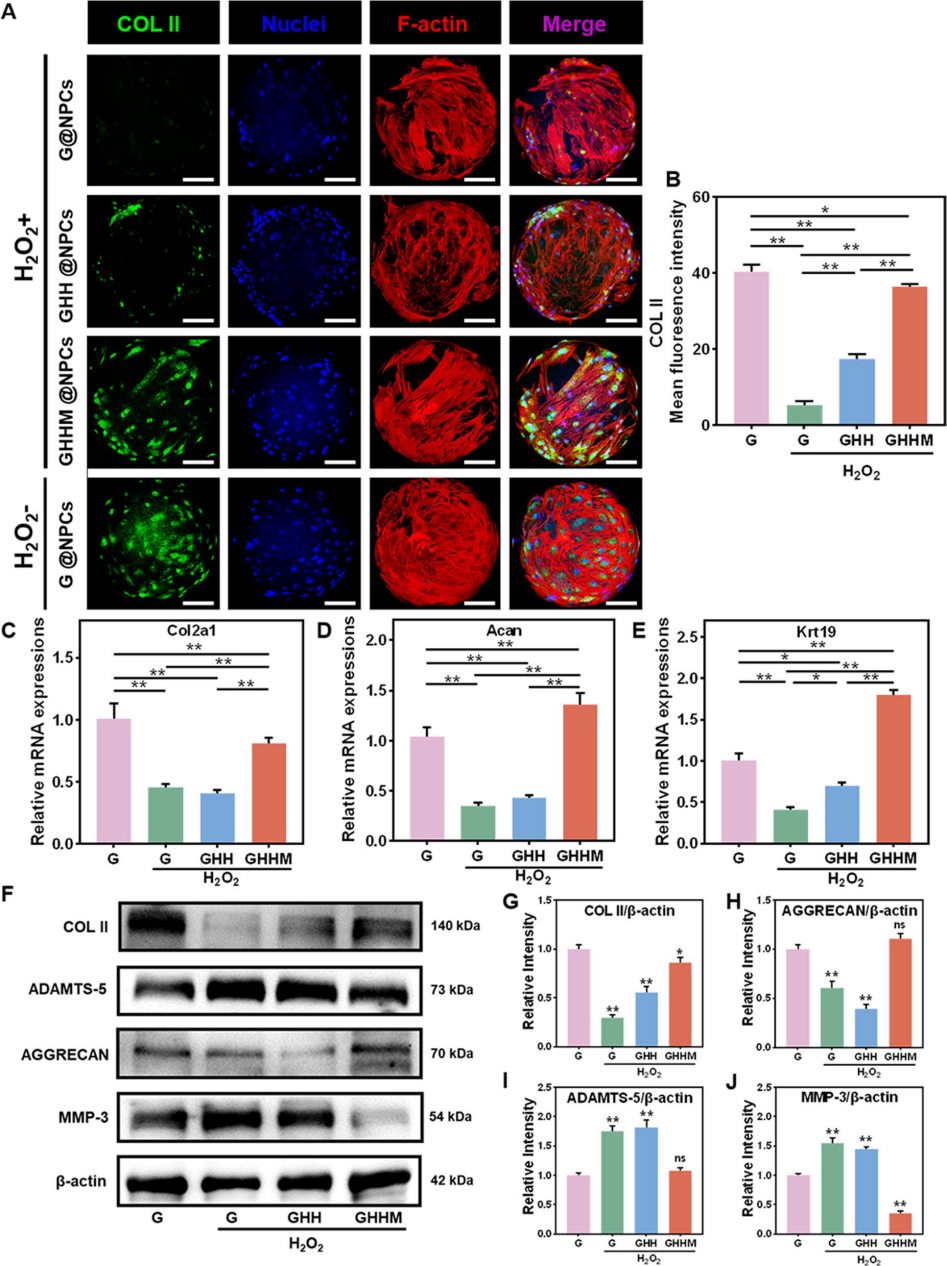
GHHM maintains the L-NPCs original phenotype in vitro. **A** Immunofluorescence analysis of COL II, scale bar = 50 μm. **B** Quantitative analysis of mean fluorescence intensity of COL II. **C**–**E** RT-qPCR analysis of the relative mRNA expression of the L-NPCs phenotype-related genes after different treatment. **F** Western blot of ECM degrading enzymes and ECM proteins in L-NPCs treated with H_2_O_2_. **G**–**J** Relative protein quantification of grey scale value for phenotype-related proteins (ratio to β-actin), compared with the control group. *p < 0.05, **p < 0.01, *ns* no significant difference

To examine if the L-NPCs would lose their original cellular bio-characteristics in the degenerative microenvironment, Collagen II (COL II) staining was performed. The results showed that, in the H_2_O_2_ treated condition, only GHHM retained the L-NPCs with high Collagen II expression level as that in the physical status (Fig. 5A, B). RT-qPCR and WB analysis also revealed the expression of the nucleus pulposus phenotype-related genes, Col2a1, Acan and Krt19, in H_2_O_2_-treated G and GHH groups were significantly lowered than that in the control group, while it was well maintained or even up-regulated in the GHHM group (Fig. 5C–J). In addition, GHHM reduced the excessive ECM catabolism caused by MMP3 and ADAMTS-5 (Fig. 5I, J). These findings suggest that GHHM microspheres maintain the metabolism and catabolism balance of the nucleus pulposus ECM, as well as the homeostasis of the L-NPCs.

### GHHM@NPCs rescues the surrounding NPCs (S-NPCs) from degeneration

**Fig. 6:**
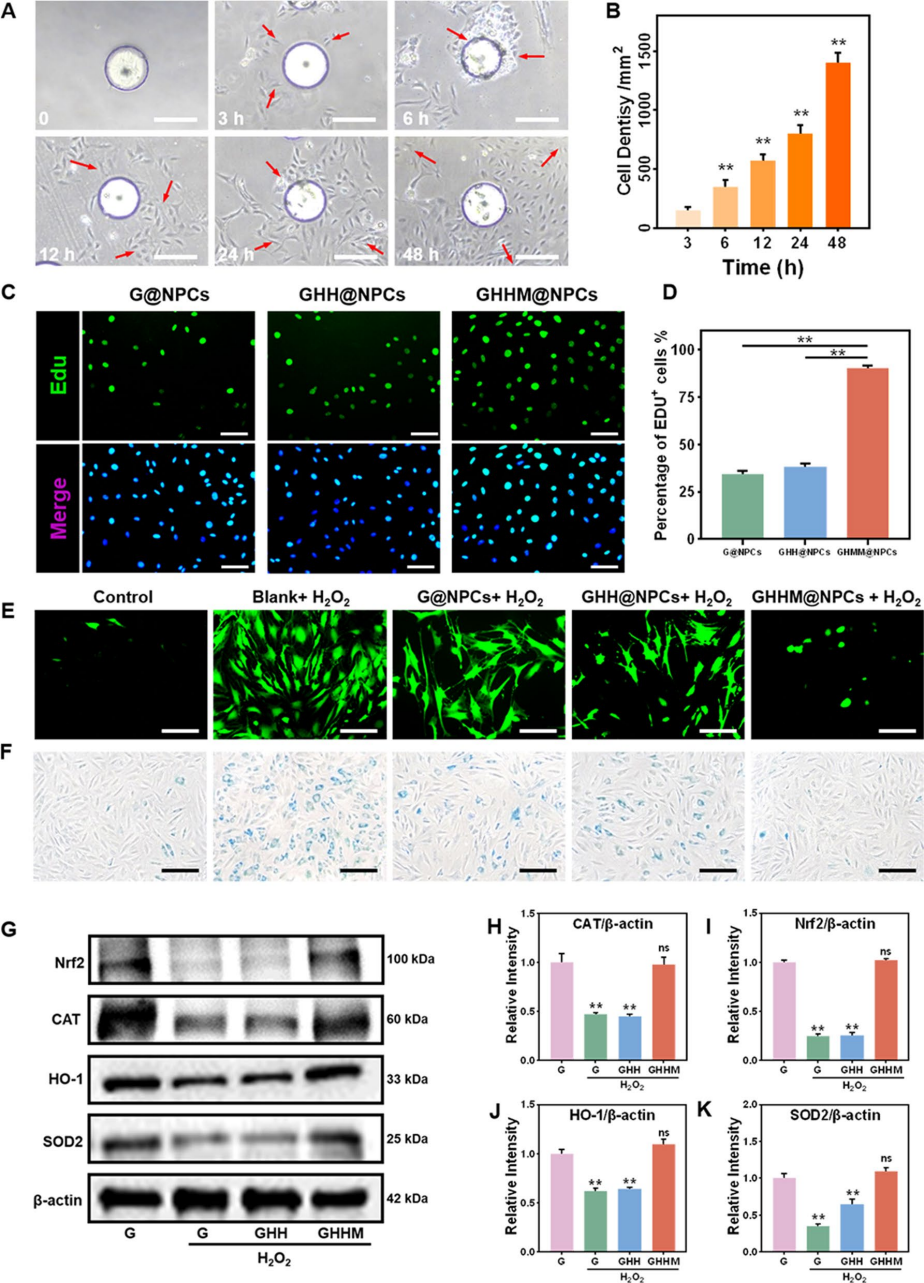
GHHM@NPCs rescues the S-NPCs from degeneration in vitro. **A** Proliferation of L-NPCs in 96-well plates, scale bar = 200 μm. **B** Quantitation of cell densities (compared with 3 h). **C** EDU staining of S-NPCs after co-culture with GHHM@NPCs in 200 μM H_2_O_2_, scale bar = 50 μm. **D** Quantitation of the ratio of EDU^+^ cells in different groups. **E** ROS analysis of S-NPCs in each group after 200 μM H_2_O_2_ treatment, scale bar = 100 μm. **F** The SA-β-Gal staining images of S-NPCs in each group after 200 μM H_2_O_2_ treatment, scale bar = 200 μm. **G** Western blot of ROS-related proteins in S-NPCs treated with different groups. **H**–**K** Relative protein quantification of grey scale value for ROS-related proteins (ratio to β-actin), compared with the control group. *p < 0.05, **p < 0.01, *ns* no significant difference

To investigate the impact of GHHM@NPCs on the surrounding environment, we first placed GHHM@NPCs directly into 96-well plates, in which the surrounding areas were pictured after 0, 3, 6, 12, 24 and 48 h. It was observed that the NPCs on the GHHM showed the ability to continuously grow and proliferate to the surrounding environment (Fig. 6A, B). After that, we went on to explore whether GHHM@NPCs could rescue the surrounding NPCs from degeneration in vitro. The GHHM@NPCs were then co-cultured with S-NPCs within the H_2_O_2_ environment for 3 days. The cell proliferation and ROS level in the surrounding microenvironment were detected by EDU and ROS staining. EDU staining showed the GHHM@NPCs significantly promoted the proliferation of the S-NPCs than the other two groups (Fig. 6C, D). At the meantime, the ROS level of the S-NPCs was found to lower in the GHHM@NPCs group (Fig. 6E, Additional file 1: Figure S5), as well as the reduced senescent cells number of the S-NPCs in the GHHM@NPCs group as revealed by the senescence-associated β galactosidase (SA-β-Gal) kit (Fig. 6F, Additional file 1: Figure S9). The expression levels of ROS-related proteins, including SOD2, Nrf2, HO-1 and CAT, in S-NPCs were evaluated by WB, which also reduced significantly in the GHHM@NPCs group (Fig. 6G–K). Based on these results, it was demonstrated the ability of GHHM@NPCs to proliferate L-NPCs and rescue the surrounding NPCs in the degenerative environment in vitro.

**Fig. 7:**
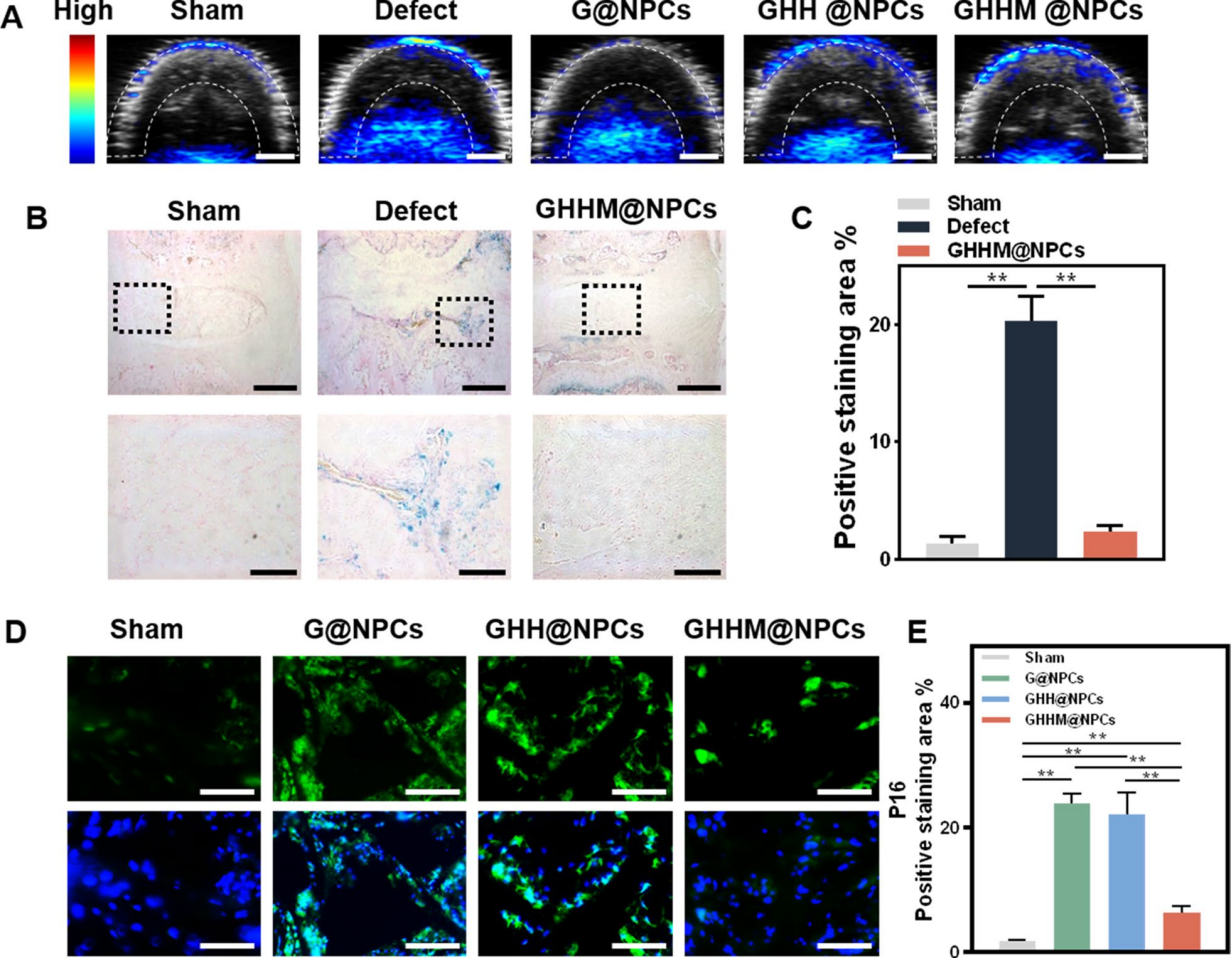
GHHM@NPCs rescues the S-NPCs from degeneration in vivo. **A** PA images of nucleus pulposus tissue 7 days after injection of different groups, scale bar = 2 mm. **B** The SA-β-Gal staining images of rat intervertebral discs after treatment by different groups, scale bar above = 1 mm, below = 300 μm. **C** Quantitative analysis of SA-β-Gal staining of rat intervertebral discs. **D** Immunofluorescence analysis of P16 in nucleus pulposus, scale bar = 200 μm. **E** Quantitative analysis of mean fluorescence intensity of P16. **p < 0.01

To further explore the ability of GHHM@NPCs to rescue the S-NPCs from degeneration in vivo, the cell-laden microspheres were injected into the degenerative nucleus pulposus (NP) of the needle-punctured rat model (Additional file 1: Figure S7A, B). The rats were divided into five groups, including Sham, Defect, G@NPCs, GHH@NPCs and GHHM@NPCs groups. Seven days after puncturing, the ROS-scavenge capacity of materials in vivo was measured by photoacoustic (PA) imaging. It showed the ROS-level in the Defect group was substantially higher than that in the Sham group, while no obvious difference was observed in the Defect, G@NPCs and GHH@NPCs groups. Remarkably, GHHM@NPCs greatly reduced ROS levels in the nucleus pulposus as compared with that in the other groups (Fig. 7A and Additional file 1: Figure S6). The SA-β-Gal kit revealed a significant decrease in the number of senescent cells in the GHHM@NPCs group in vivo (Fig. 7B, C). Meanwhile, IF staining of P16 in vivo showed higher expression in the G@NPCs and GHH@NPCs groups, and significantly lower its expression in the GHHM@NPCs group (Fig. 7D, E). The results above suggest that the GHHM@NPCs obtain excellent anti-ROS and anti-senescence capacity to rescue the S-NPCs from degeneration in IVDD.

### GHHM@NPCs inhibited IVDD progression in vivo

**Fig. 8:**
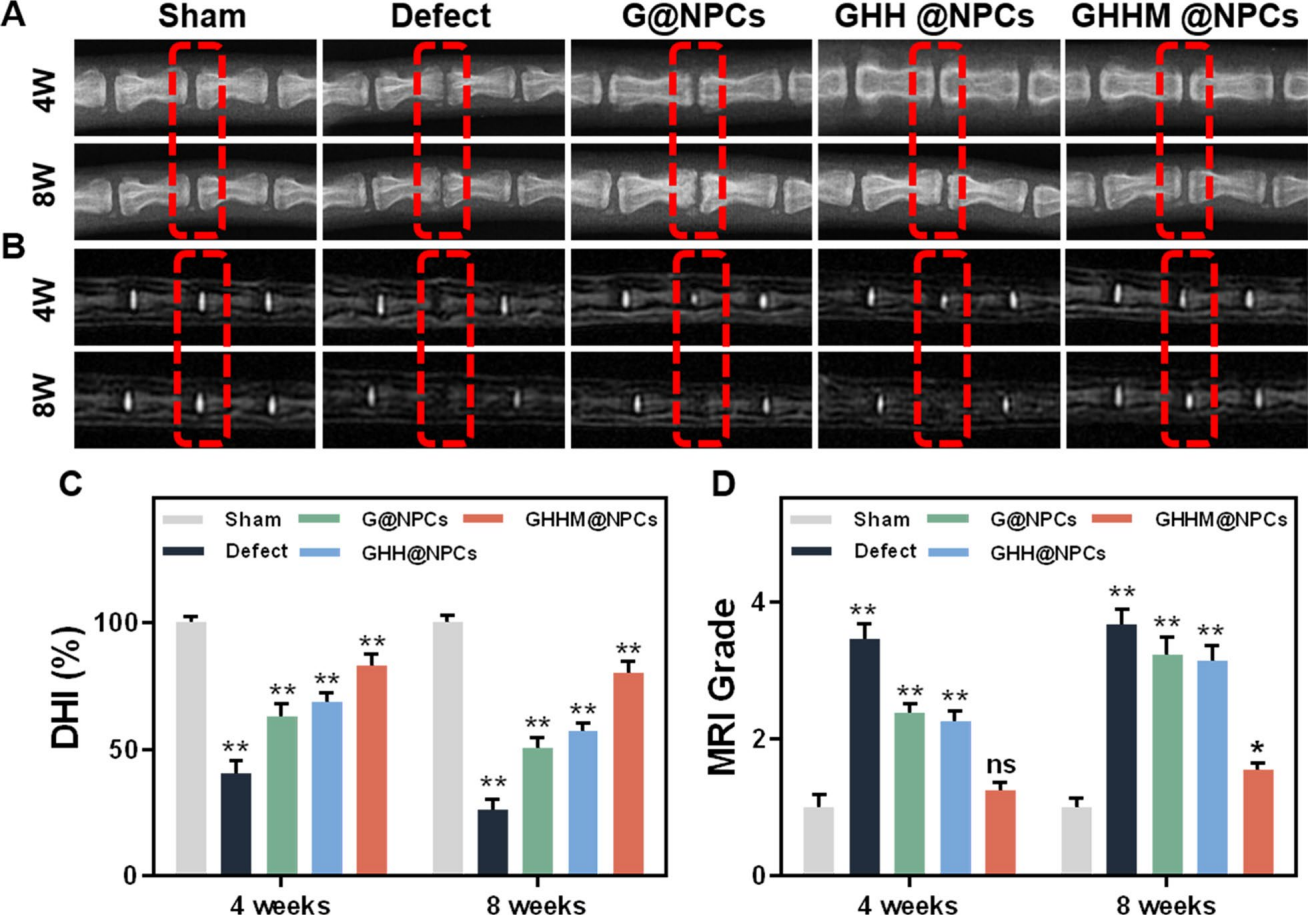
Radiological data of animal experiments. **A** Representative X-ray images of the caudal vertebrae of rats at 4 and 8 weeks (marked by red wireframe). **B** Representative MRI images of the caudal vertebrae of rats. **C** Changes in DHI at 4 and 8 weeks postoperatively (compared with the Sham group). **D** Changes in MRI grading at 4 and 8 weeks postoperatively (compared with the Sham group). *p < 0.05, **p < 0.01, *ns* no significant difference

The above experiments have shown that GHHM keeps the L-NPC active, which also improves the viability of the S-NPCs. Then, the potential therapeutic effect of GHHM@NPCs for IVDD treatment in vivo was tested. Since intervertebral space and water content of the intervertebral disc are key indicators for IVDD, magnetic resonance imaging (MRI) analysis and X-ray were firstly carried out to assess and analyze the degeneration of the rat intervertebral disc. Disc height reflects the change of IVD space and the degree of disc degeneration and lower disc height index (DHI) indicates more severe disc degeneration. As shown in Fig. 8A, the Defect group experienced a rapid collapse of the intervertebral space at 4 weeks after needle puncture, as well as the G@NPCs and GHH@NPCs groups. The continued decrease in intervertebral space at 8 weeks after treatments suggested the poor therapeutic effect of the G@NPCs and GHH@NPCs. Based on MRI scans, in which the higher T2-weighted signal indicates more abundant water content in the nucleus pulposus tissue. As showed by the MRI data, the T2-weighted signal in the discs of the Defect, G@NPCs and GHH@NPCs groups were declined after 4 weeks puncture, and it was even worse after 8 weeks. While, on the contrary, the GHHM@NPCs group well maintained the disc in a high T2-weighted signal even after 8 weeks (Fig. 8B). Moreover, disc degeneration can be graded on routine T2-weighted MRI scans using the grading system and algorithm [38]. As a result, DHI changes (Additional file 1: Figure S8) and grade analysis all revealed a significant decrease in the degeneration degree after GHHM@NPCs treatment as compared to the Defect, G@NPCs and GHH@NPCs groups (Fig. 8C, D). These findings implied the GHHM@NPCs exerted ideal effect in alleviating the degeneration of nucleus pulposus.

**Fig. 9:**
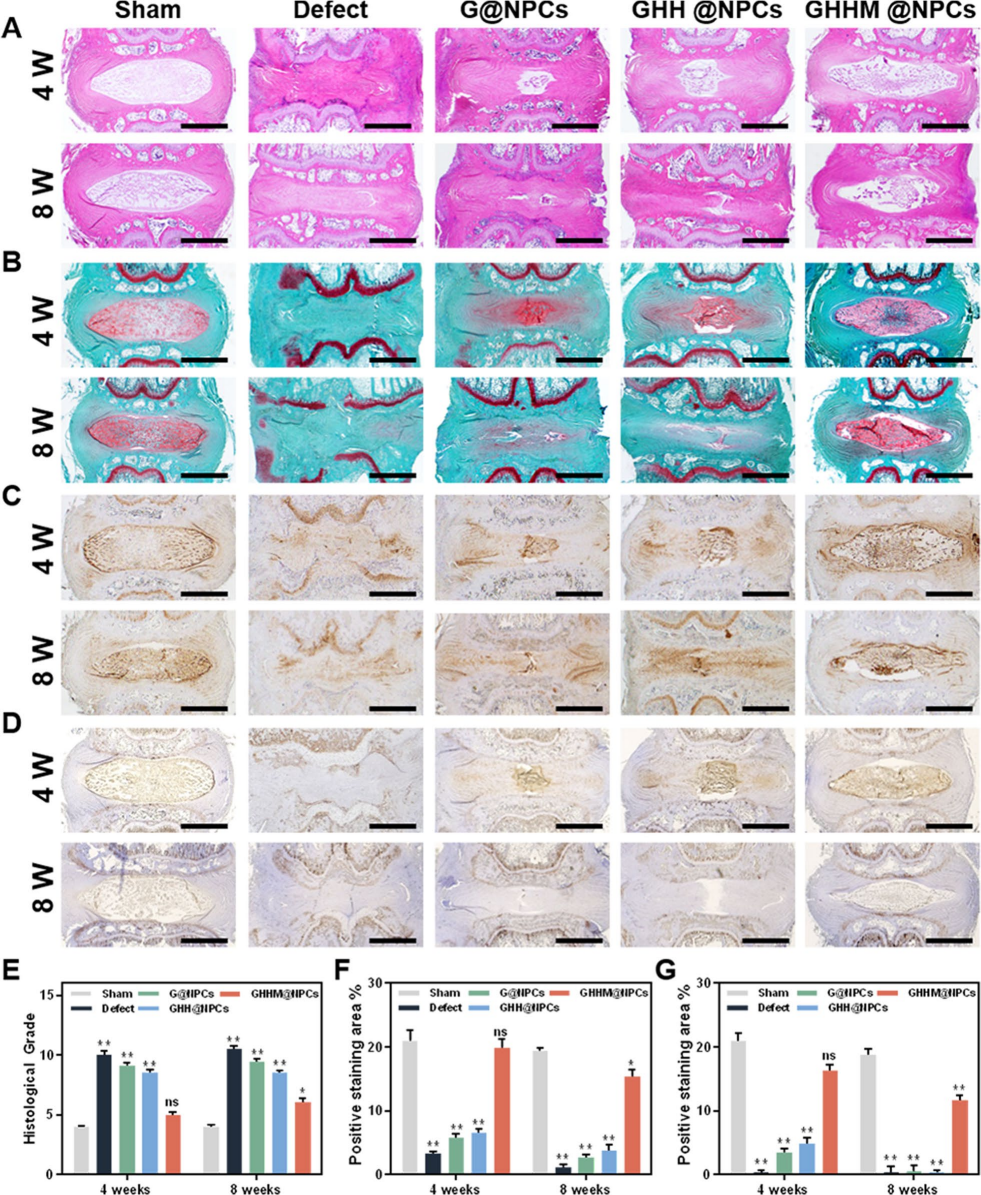
Histological evaluation of animal experiments. **A** The H&E staining images of rat intervertebral discs in each group at 4 and 8 weeks, scale bar = 1 mm. **B** Safranin-O/Fast Green staining images, scale bar = 1 mm. **C** Immunohistochemical staining images of Aggrecan at 4 and 8 weeks, scale bar = 1 mm. **D** Immunohistochemical staining images of Collagen II at 4 and 8 weeks, scale bar = 1 mm. **E** Changes in histological grading of each group at 4 and 8 weeks (compared with the Sham group). **F**, **G** Quantitative analysis of immunohistochemical staining for Aggrecan and Collagen II at 4 and 8 weeks (compared with the Sham group). *p < 0.05, **p < 0.01, *ns* no significant difference

In addition to the radiographic assessment, the pathological progress of IVDD were observed by histological examination. Hematoxylin and Eosin (H&E) staining revealed the clear architecture of the nucleus pulposus tissue and the structure of the surrounding fibrous ring in the GHHM@NPCs group at 8 weeks after puncture. The nucleus pulposus tissue in the Defect group was completely absent, and it was significantly reduced until to completely disappear at 8 weeks after puncture in the G@NPCs and GHH@NPCs groups (Fig. 9A). Safranin O fast green (SO/FG) staining revealed the proteoglycan (red) and collagen (green) content of the IVD in the GHHM@NPCs group exhibited similar morphology as that in the Sham group, manifesting abundant proteoglycans in the nucleus pulposus. In contrast, the proteoglycans were replaced by collagen in the other three groups at 8 weeks after puncture, which was accompanied by proteoglycan loss and disc collapse. G@NPCs and GHH@NPCs treatment delayed the progress of IVDD slightly but did not reverse this process (Fig. 9B). Moreover, immunohistochemistry (IHC) staining revealed the expression of Collagen II and Aggrecan in the nucleus pulposus tissue, which was consistent with H&E and SO/FG staining results (Fig. 9C, D). The quantitative analysis reflected the similar results as that in the images (Fig. 9E–G). The IHC staining results showed that MMP13 expression was significantly higher in the defect group and slightly lower in the G/GHH group, but the expression level of MMP13 in the GHHM group was significantly lower than the other groups (Additional file 1: Figure S10). The GHHM microsphere system played a positive role in reducing metalloproteases as well as helping the remodeling of ECM in the NP tissue. The results above confirm that the GHHM@NPCs have the ability to rescue the nucleus pulposus tissue from degeneration and structural damage, and further promote its regeneration.

## Discussion

Multiple activities were found to play roles in IVDD progression, including overactive inflammatory microenvironment, oxidative stress, and the imbalance between anabolic and catabolic ECM in NPCs [9, 39, 40]. All of the above interacting adverse factors make the IVD microenvironment enter into a vicious cycle. Recent studies have tried to find an approach by delivering drugs with the function of anti-inflammatory, antioxidant or growth factors into IVD directly, which successfully slow down the process of IVDD [8, 13, 17]. However, they failed to re-boost the nucleus pulposus regeneration as the lack of NPCs sources. Considering the special structure of the intervertebral disc, cell transplantation is an ideal strategy. However, the degenerative microenvironment makes it difficult for the transplanted NPCs to survive, which is a key problem that confuse us. In this study, we used Mg^2+^ to increase the ability of cell survival and proliferation rate of the NPCs, which may fight against the harsh microenvironment in the degenerative IVD. Studies have shown that Mg^2+^ is a key cofactor for enzyme activation and has been implicated in the process of regulating cellular ROS and inflammation levels [41, 42]. Furthermore, recent research work has shown that the anti-inflammatory and antioxidant properties of Mg^2+^ help in the treatment of inflammatory diseases [43–45]. In according to these, we observed that the Mg^2+^-charged NPCs exhibited satisfactory anti-ROS, anti-inflammatory, and anti-senescence abilities both in vitro and in vivo.

We used hydrogel microspheres as a carrier to make cell transplantation injectable and better simulate the three-dimensional environment of the IVD than traditional hydrogels [37, 46]. GelMA is a commonly used light-crosslinked biomaterial made from gelatin and methacrylic anhydride that show good biocompatibility and high strength [35, 47]. Hyaluronic acid (HA) is a polysaccharide presenting in cartilage and intervertebral disc tissues, and is sensitive to chemical changes [48–51]. Zhou et al. [52] used HA and GelMA double-network hydrogels as 3D printing inks for skin regeneration. Nevertheless, microspheres of other materials such as PLGA may result in denaturation of growth factors, and the acidic degradation products of PLGA may worse the poor microenvironment in IVD [53, 54]. Henry et al. developed a biphasic injectable hydrogel system that serves as a vehicle for sustained release of TGF-β1 and GDF-5, allowing their sustained release to prevent the growth factors from degradation during the treatment of disc degeneration [55]. Liu et al. [56] co-developed a novel injectable nanozyme-functionalized hyaluronic acid microspheres designed to provide a suitable microenvironment for disc tissue repair and regeneration through local lactate depletion synthesis. These methods for delivering exogenous bioactive factors and drugs to improve the intervertebral disc microenvironment have achieved some success. However, the disadvantages such as easy inactivation of bioactive factors, high cost, and lack of cellular sources for nucleus pulposus tissue repair are also exists. Francisco et al. tried to grow immature porcine NPCs in polyethylene glycol-laminin hydrogels, which was found to promote NPCs aggregation and glycosaminoglycan synthesis. Xia et al. [57] synthesized a polymeric gelatin microsphere for sustained release of growth and differentiation factor-5 (GDF-5) and as a cell delivery vehicle for NPCs, which showed good ability to regenerate IVD in vivo. Most of the previous studies focused on removing inflammatory factors, however, the implanted materials may introduce additional deleterious factors to the IVD. Alternatively, the cellular sources for regeneration of the nucleus pulposus tissue are ignored. Our study improves the microenvironment of the IVD while transplanting vibrant nucleus pulposus cells to provide a strong drive for nucleus pulposus regeneration.

Mg^2+^ is an essential mineral ion that has multiple effects and would not be inactivated in the harsh environments. Carmen et al. [58] revealed that increase the concentration of Mg^2+^ in dialysis fluids protected immune cells from oxidative stress and damage. Li et al. [45] developed a magnesium micromotor as a hydrogen generator that can act as a ROS and inflammatory scavenger, alleviating oxidative stress and reducing the levels of inflammatory cytokines. Meanwhile, Xie et al. [59] constructed an Mg^2+^/polydopamine composite hydrogel for accelerating wound healing in infected wounds. With the evidence above, Mg^2+^ are potential to play as a rosy supplier for cell energy. In our experiments, we demonstrated that Mg^2+^ bonded hydrogel microspheres system could continuously charge the L-NPCs to resist ROS-induced inflammation and senescence, which also modified the surrounding microenvironment to promote NPCs regeneration. Therefore, magnesium ions are more applicable than other bioactive factors and drugs, which can act in a complicated microenvironment. GHHM microspheres, as an injectable hydrogel scaffold, have great potential to deliver NPCs in vivo. The GHHM@NPCs increase the viability of the L-NPCs and provide a source of cells for regenerating nucleus pulposus tissue. This transplantation system continuously rescues and improves the intervertebral disc microenvironment by reducing the oxidative stress and inflammation levels to achieve the goal of repairing the degenerated nucleus pulposus tissue.

## Conclusion

In conclusion, we established a keep-charging system with Mg^2+^-bounded double-network hydrogel microspheres. The GHHM@NPCs showed strong anti-ROS, anti-inflammatory and anti-senescence ability, which efficiently regenerate the degenerative IVD both in vivo and in vitro. This cell transplantation approach provides a promising therapeutic option for the management of IVDD.

## Materials and methods

### Main materials

Gelatin methacryloyl (GelMA, EFL-GM-60) and Lap photo-initiator were obtained from Suzhou Intelligent Manufacturing Research Institute (Suzhou, China). Hyaluronic acid (HA) and L-histidine (His) were purchased from Aladdin (Shanghai, China). Magnesium chloride was purchased from Sigma-Aldrich (USA). All reagents used were of at least ACS grade.

### Preparation of HA-His

1 g of HA was dissolved in 100 ml of deionized water, 1-(3-Dimethylaminopropyl)-3-ethylcarbodiimide hydrochloride (EDC) and N-Hydroxy succinimide (NHS) were added to activate the carboxyl groups, and 400 mg of His was added to the HA solution under stirring. After passing helium gas in the conical flask, the reaction was then carried out at room temperature for 24 h. The resulting solution was dialyzed with deionized water for 5 days. The dialysis bags (molecule weight cut-off of 3.5 kDa) were purchased from Shanghai yuanye Bio-Technology Co., Ltd. After completion of dialysis, the solution was frozen at −80 °C, followed by lyophilization in a freeze dryer and prepared for use.

### Preparation of GelMA/HA-His-Mg^2+^ microspheres

We prepared GelMA/HA-His-Mg^2+^ microspheres using a microfluidic device as shown in our previous study [60]. The microfluidic device was connected to a coaxial structure syringe with internal and external dimensions of 30 G and 21 G. The oil phase was light oil (Beyotime, China), and 5% Span 80 (Aladdin, China) was added as a surfactant for the microspheres to make the microsphere structure more stable. The 10 wt % of GelMA and 2% HA-His were mixed thoroughly, and 0.5% LAP was added to the solution, which was used as the aqueous phase. Different shear forces were obtained by adjusting the appropriate flow rate ratios of oil and aqueous phases to form uniform droplets of GelMA/HA-His (GHH), and the solid GHH microspheres were formed by irradiation at the outlet with ultraviolet (UV) light at a wavelength of 405 nm. We collected the microspheres and washed them repeatedly with 75% ethanol and acetone in order to remove mineral oil and surfactants. To prepare GelMA/HA-His-Mg^2+^ (GHHM) microspheres, we used 50 mM magnesium chloride solution as a solvent and prepared composite microspheres using the same method. Then, the microspheres were washed using phosphate buffered saline (PBS) to remove the unbound magnesium ions. Finally, the washed microspheres were frozen at −80 °C and then freeze-dried for 48 h.

### Characterization of composite microspheres

The physicochemical properties of GelMA, GelMA/HA-His and GelMA/HA-His-Mg^2+^ microspheres were characterized. The surface morphology of the composite microspheres before and after lyophilization was observed using a bright-field field microscope (Zeiss Axiobert 200, USA) and a scanning electron microscope (SEM, FEI Scios 2 HiVac, USA). The diameter distribution of 100 microspheres at different flow rates in coaxial were measured and analyzed using Image J software (NIH, Bethesda, USA). SEM Mapping (FEI Scios 2 HiVac, USA) was used to analyze the surface elements of the microspheres, and EDS (Thermo Scientific, USA) was used to detect the surface composition of the composite microspheres. Fourier transform infrared spectroscopy (FTIR, Thermo Scientific Nicolet iS20, USA) was used to characterize whether HA-His was successfully synthesized.

### Magnesium ion release

To evaluate the Mg^2+^ release behavior, we packed GelMA/HA-His-Mg^2+^ microspheres (10 mg) in dialysis bags, immersed in PBS (10 ml), and incubated at 37 °C for 21 days. At specific time points (1, 2, 3, 5, 7, 9, 11, 13, 15, 17, 19 and 21 days), 5 mL of supernatant was taken, diluted to 10 mL with PBS, and then analyzed by inductively coupled plasma emission spectroscopy (ICP-OES, Thermo Fisher iCAP PRO, USA). Three parallel measurements were performed to perform averaging. To continue the release, fresh PBS (5 mL) was replenished and the system was kept at 37 °C for a longer period of time. Finally, the Mg^2+^ release curve was completed.

### Swelling and degradation rate

Swelling rate: 5 mg of microspheres were placed in a cell strainer (70 μm), placed in a 6-well plate and 5 ml of PBS was added, removed at 0, 1, 2, 4, 6, 8, 10 and 12 h, dried and weighed (Wt). The weight after immersion in PBS was W0, and the swelling rate was calculated using the formula Wt/W0. The measurements were repeated three times for each time point for three samples.

Degradation rate: 5 mg of microspheres were placed in a 15 ml centrifuge tube with 10 mL of PBS and incubated at 37 °C in a thermostat. After 0.5, 1, 2, 3, 4, 5, 6, 7, 8 weeks the microspheres were weighed after lyophilization (Wt), the total initial mass of microspheres was W0, and the remaining weight (%) was calculated using the formula Wt/W0. Measurements were repeated three times at each time for three samples.

### Biocompatibility and composite microspheres loading NPCs

The biocompatibility of the composite microspheres was investigated using rat nucleus pulposus cells (NPCs). 1 mg of composite microspheres were spread over the bottom of 96-well plates, 5 × 10^3^ NPCs were added to each well, and the appropriate amount of medium was added and incubated for 1, 3, and 7 days before further detection of cytotoxicity using the CCK-8 assay kit (Dojin Laboratories, Kumamoto, Japan). To assess the viability of NPCs on different microspheres, live/dead cell viability assays were performed using the Live/Dead Cell Staining Kit (Inbitrogen, USA). After co-culture of the composite microspheres with cells for 5 days, the NPCs on the composite microspheres were stained for skeleton and the microsphere complex was evaluated using a confocal laser microscope (LSCM, LSM800, Zeiss, Germany). In addition, 1 mg of previously prepared GHHM microspheres was weighed and added to 1 ml of complete medium and mixed well. It was equally distributed into a 96-well plate with 100 μL per well. Then 5 × 10^4^ NPCs were added to each well of a 96-well plate and co-cultured for one day to obtain GHHM@NPCs.

### EdU staining

1 × 10^4^ NPCs were seeded in 48-well plates, and appropriate amounts of G@NPCs/GHH@NPCs /GHHM@NPCs were added to co-culture, respectively. The cells were incubated for 12 h in 1X EdU working solution according to the EDU kit BeyoClick^™^ EdU Cell Proliferation Kit, followed by the preparation of Click Additive Solution to stain the proliferating cells, and finally the cells were stained using Hoechst 33,342 was used to stain the nuclei.

### ROS staining

The NPCs were co-cultured with G@NPCs, GHH@NPCs and GHHM@NPCs using 200 μM H_2_O_2_ for 3 days, and the composite microspheres were collected into 1.5 ml centrifuge tubes and stained for ROS using Reactive Oxygen Species Assay Kit (S0033S, Beyotime).

### SA-β-Gal staining

1 × 10^4^ NPCs were seeded in 48-well plates with appropriate amounts of G@NPCs/GHH@NPCs /GHHM@NPCs, respectively, and induced with 200 μM H_2_O_2_ for 3 days. The cells at the bottom of the well plates were stained for SA-β-Gal using senescence-associated β-galactosidase Kit. For SA-β-Gal staining of rat IVDD paraffin sections, sections were stained using SA-β-Gal Kit after dewaxing and hydration, and finally stained with nuclear fast red as the background color.

### RT-qPCR

Real-time quantitative PCR (RT-qPCR) was used to detect the expression of Col2a1, Acan, Krt19, TNF-α, IL-1β and IL-6. Gapdh was the reference gene. Briefly, NPCs and G@NPCs/GHH@NPCs /GHHM@NPCs were co-cultured for 3 days and then 200 μM H_2_O_2_ was added to the culture system for 5 days. Next, total RNA was extracted from the NPCs using TRIzol reagent (Invitrogen, Carlsbad, CA, USA), and then the concentration of RNA was measured using a NanoDrop 2000 spectrophotometer (Thermo Fisher Scientific, Waltham, MA, USA). Immediately thereafter, cDNA was synthesized from the extracted mRNA by reverse transcription. cDNA was synthesized from the extracted mRNA by reverse transcription reaction. Next, RT-PCR was performed using the ABI Step One Plus real-time PCR system (Applied Biosystems, USA) and SYBR Green RT-PCR kit (Takara, Japan). Each sample was repeated 3 times, and all the above experiments were done according to the reagent instructions. Additional file 1: Table S1 shows the primer sequences for each gene. The relative mRNA expression of each gene was normalized to the housekeeping gene Gapdh and analyzed using the 2^–ΔΔCt^ method.

### Western blot

Western Blot was used to determine the expression of genes associated with ROS and Cellular senescence. To detect the concentration of total protein after cell lysis, the BCA protein kit (Beyotime, P0012, China) was used. Sample proteins were then subjected to SDS electrophoresis and transferred to PVDF membranes (0.45 μm, Millipore, USA). PVDF membranes were treated with 5% BSA for 1 h to block non-specific binding of proteins. The relevant primary antibodies are presented in Additional file 1: Table S2. After PVDF membranes were washed 3 times, the membranes were incubated with the corresponding secondary antibody (1:1000) for 1 h. After incubation, the secondary antibody is washed 3 times with TBST to remove the unbound secondary antibody. Unbound secondary antibodies are removed and then detected and imaged with the chemiluminescence system. The system was used for detection and imaging. Finally, antigen-quantitative analysis of antigen–antibody complexes is performed using ImageJ software.

### Isolation and culture of rat NPCs

NPCs were isolated from the caudal intervertebral discs of SD rats (6–8 weeks old) and cultured in F12 modified Eagle’s medium (DMEM/F12, HyClone, USA) supplemented with 10% fetal bovine serum (FBS, HyClone, USA). Briefly, the caudal vertebrae of four rats were isolated under aseptic conditions and the nucleus pulposus tissue was extracted. The nucleus pulposus tissue was then digested in DMEM/F12 with 0.1 w/v% type II collagenase (Yeasen, Shanghai, China) for 2 h at 37 °C. During digestion, samples were observed and shaken well every 30 min. And excess tissue debris was filtered through a sterile 70 μm cell filter after incubation. After centrifugation to remove the supernatant, the NPCs were resuspended in culture medium and equally divided into two 10 cm cell culture dishes with 10 ml of DMEM/F12 complete medium. The NPCs were placed in the medium and maintained at 37 °C in a humidified incubator containing 5% CO2. The culture medium of NPCs was changed every 48 h. In order to ensure the activity of the cells, we generally use the P2 generation of NPCs in the subsequent experiments. The cells were placed in a humidified incubator with 5% CO2 at 37 °C. The medium for NPCs was changed every 48 h. Finally, we can obtain about 6.5 × 107–2.5 × 108 NPCs.

### Animal models and surgical procedures

All procedures followed the NIH Guide for the Care and Use of Laboratory Animals and were approved by the Institutional Animal Care and Use Committee of Soochow University. Adult male rats aged 10–12 weeks and weighing approximately 350 g were purchased from the Soochow University Experimental Animal Center. The rat caudal degeneration model was established using the previously described experimental method [57]. The rats were anesthetized by intraperitoneal injection of pentobarbital, and the puncture sites were routinely disinfected and toweled. To avoid effects between adjacent degenerated segments, a 21G needle was used to pass percutaneously through the caudal 8–9 (Co8-9) segments into the middle of the NP tissue (depth controlled at 5 mm) and confirmed with X-ray (Fig. 6C). The needle was rotated 360° after passing through the annulus and held for 30 s to ensure the degenerative effect. Five groups were assigned in this study: (1) no needle penetration (sham group), (2) needle penetration and PBS injection (defect group), (3) needle penetration and injection of GelMA microspheres loaded with NPCs (G@NPCs group), (4) needle penetration and injection of GelMA-HA-His microspheres loaded with NPCs (GHH@NPCs group), (5) needle penetration and injection of GelMA-HA-His-Mg^2+^ microspheres (GHHM@NPCs group). In animal experiments, all injections were administered by micropump. The defect group was injected with 10μL of PBS, other than that, the other experimental groups were injected with approximately 10μL microspheres (G@NPCs/GHH@NPCs/GHHM@NPCs) that contained almost no additional liquid. We set up two time points to assess intervertebral disc degeneration in the caudal spine of rats at week four and week eight, where eight rats were in each group at each time point. After surgery, the rats were transferred to a warm and ventilated environment.

### Fabrication of Lipo@HRP&ABTS nanoprobes and H_2_O_2_ detection at NP sites

Lipo@HRP&ABTS nanoprobes were prepared according to the method established in a previous study [60, 61]. Briefly, 1,2-hexadecanoyl-sn-glycero-3-phosphocholine, cholesterol, 1, 2-distearoyl-sn-glycero-3- phosphoethanolamine-N-(amino-(polyethylene glycol)-2000) (DSPE-PEG) and 2,2′-azobis-(3- ethylbenzothiazoline-6-sulfonic acid) (ABTS) were dissolved in methanol at a molar ratio of 6:4:0. 5:12. The solution was allowed to condense into a lipid film by rotary evaporation, after which 1 mL of PBS solution containing horseradish peroxidase (HRP) was added. The compounds were sonicated at 65 °C, followed by rehydration with five freeze–thaw cycles. The suspension was then squeezed 50 times through a 200 nm filter at 50 °C. An S-300 Sephadex column was used to purify the Lipo@HRP& ABTS nanoprobes. On postoperative day 7, 10 μL of Lipo@HRP&ABTS nanoprobe was injected locally into the NP tissue site. 15 min later, a layer of ultrasound gel was applied to the skin over the injured IVDs and a detector from the Vevo LAZR imaging system (FUJIFILM VisualSonics Inc.) was placed on the skin covering the injured IVDs. The level of H_2_O_2_ was then measured.

### Radiological evaluation

X-ray and MRI imaging operations were performed on the caudal spine of rats at 4 and 8 weeks postoperatively. All rats were anesthetized and placed in a supine position with their tails extended. The disc height index (DHI) measured on the X-ray images was calculated using ImageJ software for comparison between groups (DHI = twice the sum of the anterior, middle, and posterior marginal heights of the intervertebral space/sum of the anterior, middle, and posterior heights of adjacent vertebral bodies, DHI% = DHI measured at each time point/DHI of the sham group × 100%) (Additional file 1: Figure S4). T2-weighted images of the caudal spine were acquired by a 1.5 T MRI scanner (Magnetom Essenza, Siemens Medical Solution, Erlangen, Germany), and the nucleus pulposus tissue signal was measured using ImageJ software.

### Histological and Immunohistochemical analyses

Samples were collected at week 4 and week 8, respectively, and fixed in 10% formaldehyde solution for 24 h. After decalcification with 14% ethylenediaminetetraacetic acid (EDTA) for 60 days, samples were embedded in paraffin and sectioned to a thickness of 6 μm. Further, H&E staining was performed to examine the histology of the tissue. Changes and distribution of collagen and proteoglycans were assessed using safranin O-fast green (SO/FG) staining. To assess COL II and Aggrecan changes in vivo, sections were stained by immunohistochemistry after antigen retrieval. Briefly, all sections were placed in a 3% H_2_O_2_ solution and incubated without light for 15 min at room temperature after antigen retrieval. After washing 3 times with PBS, the sections were blocked with 10% donkey serum plus 0.3% Triton X-100 and 1% BSA for 2 h. Sections were incubated overnight at 4 °C with the primary antibody against COL II and Aggrecan, followed by incubation with HRP-labeled secondary antibody for 30 min at room temperature. Samples were stained with 3,3'-diaminobenzidine (DAB), and nuclei were counterstained with hematoxylin. Images were taken and observed with a light microscope. Semi-quantitative immunohistochemical stain analysis was conducted with Image J software.

### Statistical analysis

In the research, the mean ± standard deviation of three independent experiments was used to represent all of the analyzed data. SPSS 22 (SPSS Corp., Chicago, IL, USA) was used for statistics. GraphPad Prism software (GraphPad Software Inc.) was used to draw all figures. Analysis of variance within groups was used, and the t test was used to analyze differences between groups. P-values less than 0.05 were considered statistically significant. (*p < 0.05; **p < 0.01; ns, no significant difference).

### Supplementary Information

**Additional file 1: Table S1.** Primers for RT-qPCR. **Table S2.** Primary antibodies used in this study. **Figure S1.** Pictures of GHHM microspheres in EP tubes, in PBS and in mineral oil, respectively, scale bar = 100 μm. **Figure S2.** Selection of Mg^2 +^ concentration. A) Live/Dead staining of cells after treatment with different concentrations of Mg^2 + ^for 7 days, scale bar = 400 μm. B) Percentage of live cells in different concentrations of Mg^2 +^ (compared with the control group). C) CCK-8 assay of different concentrations of Mg^2 +^ (compared with the control group). **Figure S3.** Selection of H_2_O_2_ concentration. A) Live/Dead staining of cells after treatment with different concentrations of H_2_O_2_ for 3 days, scale bar = 200 μm. B) CCK-8 assay of different concentrations of H_2_O_2_ (compared with the control group). **Figure S4.** P21 immunofluorescence images after 200 μM H_2_O_2_ treatment, scale bar = 50 μm. **Figure S5.** Quantification of ROS levels in each group of S-NPCs after 200 μM H_2_O_2_ treatment. **Figure S6.** Quantification of ROS levels in nucleus pulposus tissue in situ 7 days after injection in different groups. **Figure S7.** Establishment of a rat caudal degeneration model. A) Schematic diagram of animal experimental operations. B) Pictures of intraoperative x-ray localization. **Figure S8.** Calculation formula and diagram of DHI. **Figure S9.** Quantification of the proportion of SA-β-Gal staining positive cells in each group of S-NPCs after 200 μM H_2_O_2_ treatment. **Figure S10.** Immunohistochemical staining images of MMP13 at 4 weeks, above scale bar = 1 mm, below scale bar = 200 μm.

## Data Availability

The raw data and processed data required to reproduce these findings are available from the corresponding author upon request.

## References

[1] Hoy D, March L, Brooks P, Blyth F, Woolf A, Bain C, Williams G, Smith E, Vos T, Barendregt J (2014). The global burden of low back pain: estimates from the Global Burden of Disease 2010 study. Ann Rheum Dis.

[2] Goodman DM, Burke AE, Livingston EH (2013). JAMA patient page. Low back pain JAMA.

[3] Knezevic NN, Candido KD, Vlaeyen JWS, Van Zundert J, Cohen SP (2021). Low back pain. Lancet.

[4] Morlion B (2013). Chronic low back pain: pharmacological, interventional and surgical strategies. Nat Rev Neurol.

[5] Fenton-White HA (2021). Trailblazing: the historical development of the posterior lumbar interbody fusion (PLIF). Spine J.

[6] Pan M, Li Q, Li S, Mao H, Meng B, Zhou F, Yang H (2020). Percutaneous endoscopic lumbar discectomy: indications and complications. Pain Phys.

[7] Zhang X, Hu Y, Hao D, Li T, Jia Y, Hu W, Xu Z (2022). New strategies for the treatment of intervertebral disc degeneration: cell, exosome, gene, and tissue engineering. Am J Transl Res.

[8] Risbud MV, Shapiro IM (2014). Role of cytokines in intervertebral disc degeneration: pain and disc content. Nat Rev Rheumatol.

[9] Francisco V, Pino J, Gonzalez-Gay MA, Lago F, Karppinen J, Tervonen O, Mobasheri A, Gualillo O (2022). A new immunometabolic perspective of intervertebral disc degeneration. Nat Rev Rheumatol.

[10] Gao B, Jiang B, Xing W, Xie Z, Luo Z, Zou W (2022). Discovery and application of postnatal nucleus pulposus progenitors essential for intervertebral disc homeostasis and degeneration. Adv Sci (Weinh).

[11] Hu B, Lv X, Wei L, Wang Y, Zheng G, Yang C, Zang F, Wang J, Li J, Wu X (2022). Sensory nerve maintains intervertebral disc extracellular matrix homeostasis Via CGRP/CHSY1 axis. Adv Sci (Weinh).

[12] Sun K, Jiang J, Wang Y, Sun X, Zhu J, Xu X, Sun J, Shi J (2022). The role of nerve fibers and their neurotransmitters in regulating intervertebral disc degeneration. Ageing Res Rev.

[13] Liu W, Ma Z, Wang Y, Yang J (2023). Multiple nano-drug delivery systems for intervertebral disc degeneration: current status and future perspectives. Bioact Mater.

[14] Zhou X, Shen N, Tao Y, Wang J, Xia K, Ying L, Zhang Y, Huang X, Hua J, Liang C (2023). Nucleus pulposus cell-derived efficient microcarrier for intervertebral disc tissue engineering. Biofabrication.

[15] Li Z, Cai F, Tang J, Xu Y, Guo K, Xu Z, Feng Y, Xi K, Gu Y, Chen L (2023). Oxygen metabolism-balanced engineered hydrogel microspheres promote the regeneration of the nucleus pulposus by inhibiting acid-sensitive complexes. Bioact Mater.

[16] Chen T, Qian Q, Makvandi P, Zare EN, Chen Q, Chen L, Zhang Z, Zhou H, Zhou W, Wang H (2023). Engineered high-strength biohydrogel as a multifunctional platform to deliver nucleic acid for ameliorating intervertebral disc degeneration. Bioact Mater.

[17] Lim S, An SB, Jung M, Joshi HP, Kumar H, Kim C, Song SY, Lee JR, Kang M, Han I, Kim BS (2022). Local delivery of senolytic drug inhibits intervertebral disc degeneration and restores intervertebral disc structure. Adv Healthc Mater.

[18] Sakai D, Andersson GB (2015). Stem cell therapy for intervertebral disc regeneration: obstacles and solutions. Nat Rev Rheumatol.

[19] Lyu FJ, Cheung KM, Zheng Z, Wang H, Sakai D, Leung VY (2019). IVD progenitor cells: a new horizon for understanding disc homeostasis and repair. Nat Rev Rheumatol.

[20] Chen J, Zhu H, Xia J, Zhu Y, Xia C, Hu Z, Jin Y, Wang J, He Y, Dai J, Hu Z (2023). High-performance multi-dynamic bond cross-linked hydrogel with spatiotemporal siRNA delivery for gene-cell combination therapy of intervertebral disc degeneration. Adv Sci (Weinh).

[21] Peng B, Li Y (2022). Concerns about cell therapy for intervertebral disc degeneration. NPJ Regen Med.

[22] Oehme D, Goldschlager T, Ghosh P, Rosenfeld JV, Jenkin G (2015). Cell-based therapies used to treat lumbar degenerative disc disease: a systematic review of animal studies and human clinical trials. Stem Cells Int.

[23] Huang YC, Leung VY, Lu WW, Luk KD (2013). The effects of microenvironment in mesenchymal stem cell-based regeneration of intervertebral disc. Spine J.

[24] Kass LE, Nguyen J (2022). Nanocarrier-hydrogel composite delivery systems for precision drug release. Wiley Interdiscip Rev Nanomed Nanobiotechnol.

[25] Cao H, Duan L, Zhang Y, Cao J, Zhang K (2021). Current hydrogel advances in physicochemical and biological response-driven biomedical application diversity. Signal Transduct Target Ther.

[26] Tao B, Lin C, Qin X, Yu Y, Guo A, Li K, Tian H, Yi W, Lei D, Chen Y, Chen L (2022). Fabrication of gelatin-based and Zn(2+)-incorporated composite hydrogel for accelerated infected wound healing. Mater Today Bio.

[27] Li Y, Han Y, Wang X, Peng J, Xu Y, Chang J (2017). Multifunctional hydrogels prepared by dual ion cross-linking for chronic wound healing. ACS Appl Mater Interfaces.

[28] Shi W, Fang F, Kong Y, Greer SE, Kuss M, Liu B, Xue W, Jiang X, Lovell P, Mohs AM (2021). Dynamic hyaluronic acid hydrogel with covalent linked gelatin as an anti-oxidative bioink for cartilage tissue engineering. Biofabrication..

[29] Lin S, Yin S, Shi J, Yang G, Wen X, Zhang W, Zhou M, Jiang X (2022). Orchestration of energy metabolism and osteogenesis by Mg(2+) facilitates low-dose BMP-2-driven regeneration. Bioact Mater.

[30] Maguire D, Neytchev O, Talwar D, McMillan D, Shiels PG (2018). Telomere homeostasis: interplay with magnesium. Int J Mol Sci.

[31] Yamagami R, Bingaman JL, Frankel EA, Bevilacqua PC (2018). Cellular conditions of weakly chelated magnesium ions strongly promote RNA stability and catalysis. Nat Commun.

[32] Maier JA, Castiglioni S, Locatelli L, Zocchi M, Mazur A (2021). Magnesium and inflammation: advances and perspectives. Semin Cell Dev Biol.

[33] Zheltova AA, Kharitonova MV, Iezhitsa IN, Spasov AA (2016). Magnesium deficiency and oxidative stress: an update. Biomedicine (Taipei).

[34] Wolf FI, Trapani V, Simonacci M, Ferre S, Maier JA (2008). Magnesium deficiency and endothelial dysfunction: is oxidative stress involved?. Magnes Res.

[35] Kurian AG, Singh RK, Patel KD, Lee JH, Kim HW (2022). Multifunctional GelMA platforms with nanomaterials for advanced tissue therapeutics. Bioact Mater.

[36] Gao Q, Niu X, Shao L, Zhou L, Lin Z, Sun A, Fu J, Chen Z, Hu J, Liu Y, He Y (2019). 3D printing of complex GelMA-based scaffolds with nanoclay. Biofabrication.

[37] Zhang T, Wang Y, Li R, Xin J, Zheng Z, Zhang X, Xiao C, Zhang S (2023). ROS-responsive magnesium-containing microspheres for antioxidative treatment of intervertebral disc degeneration. Acta Biomater.

[38] Pfirrmann CW, Metzdorf A, Zanetti M, Hodler J, Boos N (2001). Magnetic resonance classification of lumbar intervertebral disc degeneration. Spine (Phila Pa 1976).

[39] Lyu FJ, Cui H, Pan H, Mc Cheung K, Cao X, Iatridis JC, Zheng Z (2021). Painful intervertebral disc degeneration and inflammation: from laboratory evidence to clinical interventions. Bone Res.

[40] McHugh J (2020). Linking cell mechanobiology and inflammation in IVD degeneration. Nat Rev Rheumatol.

[41] Fiorentini D, Cappadone C, Farruggia G, Prata C (2021). Magnesium: biochemistry, nutrition, detection, and social impact of diseases linked to its deficiency. Nutrients.

[42] Glasdam SM, Glasdam S, Peters GH (2016). The importance of magnesium in the human body: a systematic literature review. Adv Clin Chem.

[43] Kwesiga MP, Gillette AA, Razaviamri F, Plank ME, Canull AL, Alesch Z, He W, Lee BP, Guillory RJ (2023). Biodegradable magnesium materials regulate ROS-RNS balance in pro-inflammatory macrophage environment. Bioact Mater.

[44] Sharikabad MN, Ostbye KM, Lyberg T, Brors O (2001). Effect of extracellular Mg(2+) on ROS and Ca(2+) accumulation during reoxygenation of rat cardiomyocytes. Am J Physiol Heart Circ Physiol.

[45] Xu C, Wang S, Wang H, Liu K, Zhang S, Chen B, Liu H, Tong F, Peng F, Tu Y, Li Y (2021). Magnesium-based micromotors as hydrogen generators for precise rheumatoid arthritis therapy. Nano Lett.

[46] Xu H, Sun M, Wang C, Xia K, Xiao S, Wang Y, Ying L, Yu C, Yang Q, He Y (2020). Growth differentiation factor-5-gelatin methacryloyl injectable microspheres laden with adipose-derived stem cells for repair of disc degeneration. Biofabrication.

[47] Yue K, Trujillo-de Santiago G, Alvarez MM, Tamayol A, Annabi N, Khademhosseini A (2015). Synthesis, properties, and biomedical applications of gelatin methacryloyl (GelMA) hydrogels. Biomaterials.

[48] Guo W, Douma L, Hu MH, Eglin D, Alini M, Secerovic A, Grad S, Peng X, Zou X, D'Este M, Peroglio M (2022). Hyaluronic acid-based interpenetrating network hydrogel as a cell carrier for nucleus pulposus repair. Carbohydr Polym.

[49] Inoue M, Isa ILM, Orita S, Suzuki-Narita M, Inage K, Shiga Y, Norimoto M, Umimura T, Sakai T, Eguchi Y (2021). An injectable hyaluronic acid hydrogel promotes intervertebral disc repair in a rabbit model. Spine (Phila Pa 1976).

[50] Yamamoto T, Suzuki S, Fujii T, Mima Y, Watanabe K, Matsumoto M, Nakamura M, Fujita N (2021). Efficacy of hyaluronic acid on intervertebral disc inflammation: an in vitro study using notochordal cell lines and human disc cells. J Orthop Res.

[51] Mohd Isa IL, Abbah SA, Kilcoyne M, Sakai D, Dockery P, Finn DP, Pandit A (2018). Implantation of hyaluronic acid hydrogel prevents the pain phenotype in a rat model of intervertebral disc injury. Sci Adv.

[52] Zhou F, Hong Y, Liang R, Zhang X, Liao Y, Jiang D, Zhang J, Sheng Z, Xie C, Peng Z (2020). Rapid printing of bio-inspired 3D tissue constructs for skin regeneration. Biomaterials.

[53] Xiao L, Gao D, Zhang Y, Liu C, Yin Z (2023). Codelivery of TGF-beta1 and anti-miR-141 by PLGA microspheres inhibits progression of intervertebral disc degeneration. J Orthop Surg Res.

[54] Cheng H, Guo Q, Zhao H, Liu K, Kang H, Gao F, Guo J, Yuan X, Hu S, Li F (2022). An injectable hydrogel scaffold loaded with dual-drug/sustained-release PLGA microspheres for the regulation of macrophage polarization in the treatment of intervertebral disc degeneration. Int J Mol Sci.

[55] Henry N, Clouet J, Fragale A, Griveau L, Chedeville C, Veziers J, Weiss P, Le Bideau J, Guicheux J, Le Visage C (2017). Pullulan microbeads/Si-HPMC hydrogel injectable system for the sustained delivery of GDF-5 and TGF-beta1: new insight into intervertebral disc regenerative medicine. Drug Deliv.

[56] Shen J, Chen A, Cai Z, Chen Z, Cao R, Liu Z, Li Y, Hao J (2022). Exhausted local lactate accumulation via injectable nanozyme-functionalized hydrogel microsphere for inflammation relief and tissue regeneration. Bioact Mater.

[57] Xia K, Zhu J, Hua J, Gong Z, Yu C, Zhou X, Wang J, Huang X, Yu W, Li L (2019). Intradiscal injection of induced pluripotent stem cell-derived nucleus pulposus-like cell-seeded polymeric microspheres promotes rat disc regeneration. Stem Cells Int.

[58] Vida C, Carracedo J, de Sequera P, Bodega G, Perez R, Alique M, Ramirez R (2021). A high magnesium concentration in citrate dialysate prevents oxidative stress and damage in human monocytes in vitro. Clin Kidney J.

[59] Guo Z, Zhang Z, Zhang N, Gao W, Li J, Pu Y, He B, Xie J (2022). A Mg(2+)/polydopamine composite hydrogel for the acceleration of infected wound healing. Bioact Mater.

[60] Zhu Z, Yu Q, Li H, Han F, Guo Q, Sun H, Zhao H, Tu Z, Liu Z, Zhu C, Li B (2023). Vanillin-based functionalization strategy to construct multifunctional microspheres for treating inflammation and regenerating intervertebral disc. Bioact Mater.

[61] Chen Q, Liang C, Sun X, Chen J, Yang Z, Zhao H, Feng L, Liu Z (2017). H(2)O(2)-responsive liposomal nanoprobe for photoacoustic inflammation imaging and tumor theranostics via in vivo chromogenic assay. Proc Natl Acad Sci U S A.

