# Supplementary material for: Transplantation of active nucleus pulposus cells with a keep-charging hydrogel microsphere system to rescue intervertebral disc degeneration
Source: J Nanobiotechnology. 2023 Nov 28;21:453. doi: 10.1186/s12951-023-02226-1 (PMC10683266; doi:10.1186/s12951-023-02226-1)
Supplement: Supplementary file 1 — Additional file 1: Table S1. Primers for RT-qPCR. Table S2. Primary antibodies used in this study. Figure S1. Pictures of GHHM microspheres in EP tubes, in PBS and in mineral oil, respectively, scale bar = 100 μm. Figure S2. Selection of Mg2 + concentration. A) Live/Dead staining of cells after treatment with different concentrations of Mg2 + for 7 days, scale bar = 400 μm. B) Percentage of live cells in different concentrations of Mg2 + (compared with the control group). C) CCK-8 assay of different concentrations of Mg2 + (compared with the control group). Figure S3. Selection of H2O2 concentration. A) Live/Dead staining of cells after treatment with different concentrations of H2O2 for 3 days, scale bar = 200 μm. B) CCK-8 assay of different concentrations of H2O2 (compared with the control group). Figure S4. P21 immunofluorescence images after 200 μM H2O2 treatment, scale bar = 50 μm. Figure S5. Quantification of ROS levels in each group of S-NPCs after 200 μM H2O2 treatment. Figure S6. Quantification of ROS levels in nucleus pulposus tissue in situ 7 days after injection in different groups. Figure S7. Establishment of a rat caudal degeneration model. A) Schematic diagram of animal experimental operations. B) Pictures of intraoperative x-ray localization. Figure S8. Calculation formula and diagram of DHI. Figure S9. Quantification of the proportion of SA-β-Gal staining positive cells in each group of S-NPCs after 200 μM H2O2 treatment. Figure S10. Immunohistochemical staining images of MMP13 at 4 weeks, above scale bar = 1 mm, below scale bar = 200 μm. [file 12951_2023_2226_MOESM1_ESM.docx]

**Additional Tables**

**Table S1.** Primers for RT-qPCR

| **Gene** | **Forward (5’-3’)** | **Reverse (5’-3’)** |
| --- | --- | --- |
| ***Gapdh*** | GACATGCCGCCTGGAGAAAC | AGCCCAGGATGCCCTTTAGT |
| ***Tnf-a*** | ACTGAACTTCGGGGTGATCG | GCTTGGTGGTTTGCTACGAC |
| ***Il-1b*** | TTGAGTCTGCACAGTTCCCC | GTCCTGGGGAAGGCATTAGG |
| ***Il-6*** | GAAAATCTGCTCTGGTCTTCTGGAG | CACTAGGTTTGCCGAGTAGACCTC |
| ***Col2a1*** | GCCAGGATGCCCGAAAATTAG | GGCTGCAAAGTTTCCTCCAC |
| ***Acan*** | ATGCCTTCCGAGCTACAGAG | GCAGGGAGTGTCCATCAGAC |
| ***Krt19*** | TCTCAGACCTGCGTCCCTTA | CGGAGGATGAGGACACGATG |

**Table S2.** Primary antibodies used in this study

| **Antibody** | **Vendor** | **Catalog number** |
| --- | --- | --- |
| **Vinculin** | Abcam | ab129002 |
| **TNF alpha** | Abcam | ab6671 |
| **Collagen II** | Abcam | ab188570 |
| **Aggrecan** | Proteintech | 13880-1-AP |
| **ADAMTS5** | Abcam | ab41037 |
| **MMP3** | Abcam | ab52915 |
| **NRF2** | Proteintech | 16396-1-AP |
| **Catalase** | Affinity | DF7545 |
| **SOD2** | Proteintech | 24127-1-AP |
| **Heme Oxygenase 1** | ZENBIO | R24541 |
| **P53** | Affinity | AF0879 |
| **P21** | Proteintech | 10355-1-AP |
| **P16** | Abcam | ab51243 |
| **Beta Actin** | Proteintech | 81115-1-RR |

**Additional Figures:**


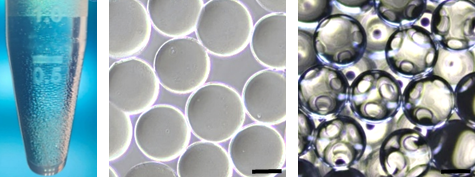


**Figure S1**. Pictures of GHHM microspheres in EP tubes, in PBS and in mineral oil, respectively, scale bar = 100μm.


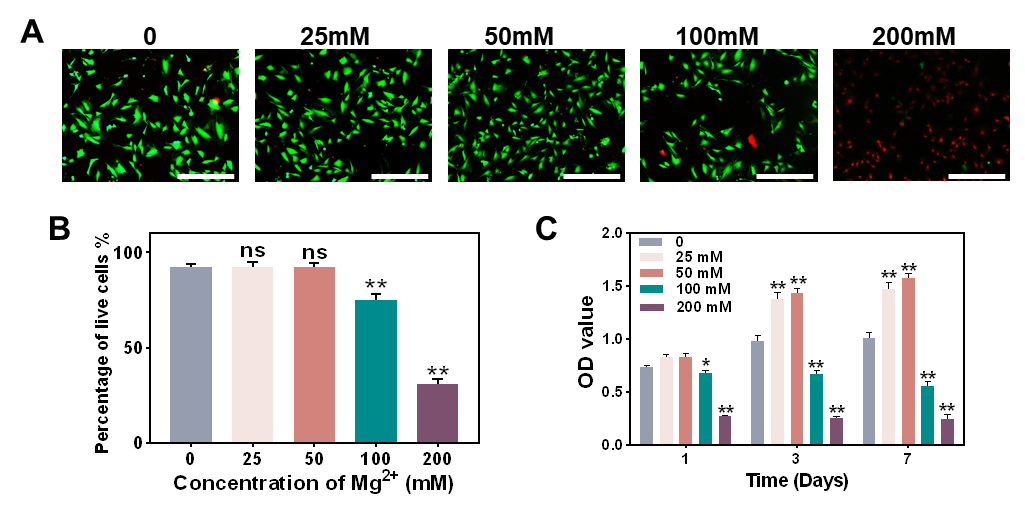


**Figure S2**. Selection of Mg^2+^ concentration. A) Live/Dead staining of cells after treatment with different concentrations of Mg^2+^ for 7 days, scale bar = 400 μm. B) Percentage of live cells in different concentrations of Mg^2+^ (compared with the control group). C) CCK-8 assay of different concentrations of Mg^2+^ (compared with the control group).


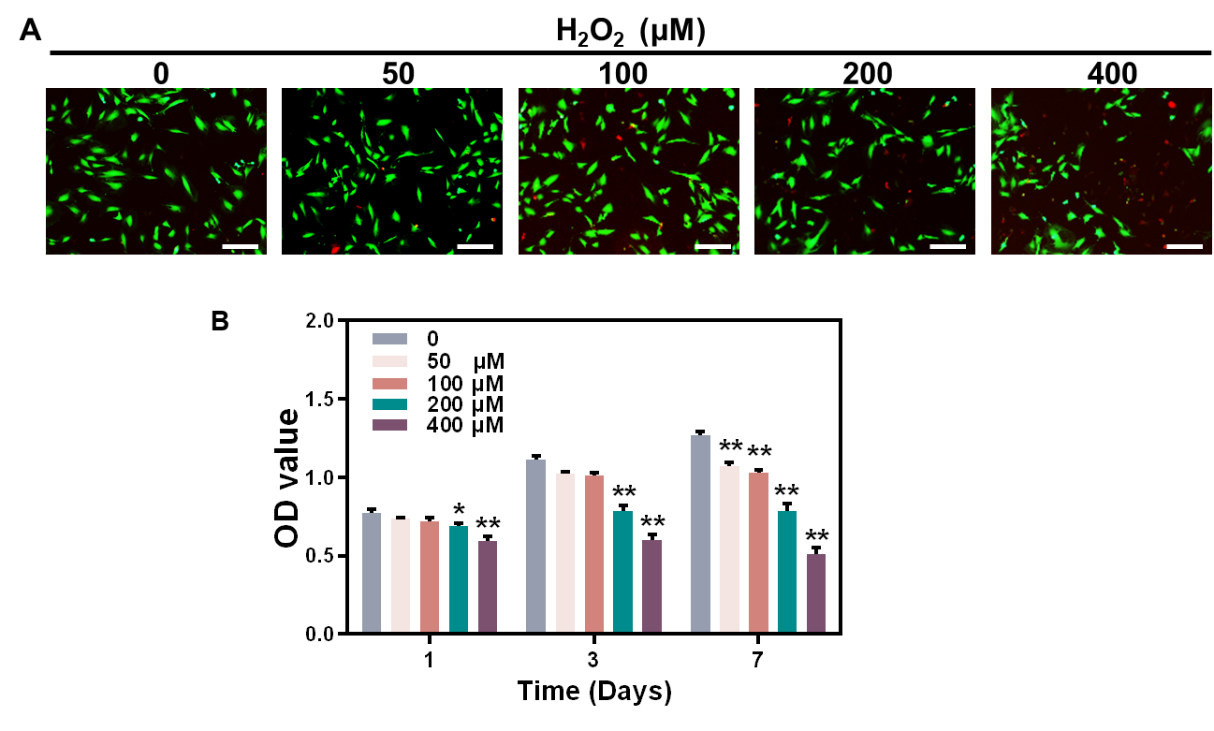


**Figure S3**. Selection of H_2_O_2_ concentration. A) Live/Dead staining of cells after treatment with different concentrations of H_2_O_2_ for 3 days, scale bar = 200 μm. B) CCK-8 assay of different concentrations of H_2_O_2_ (compared with the control group).


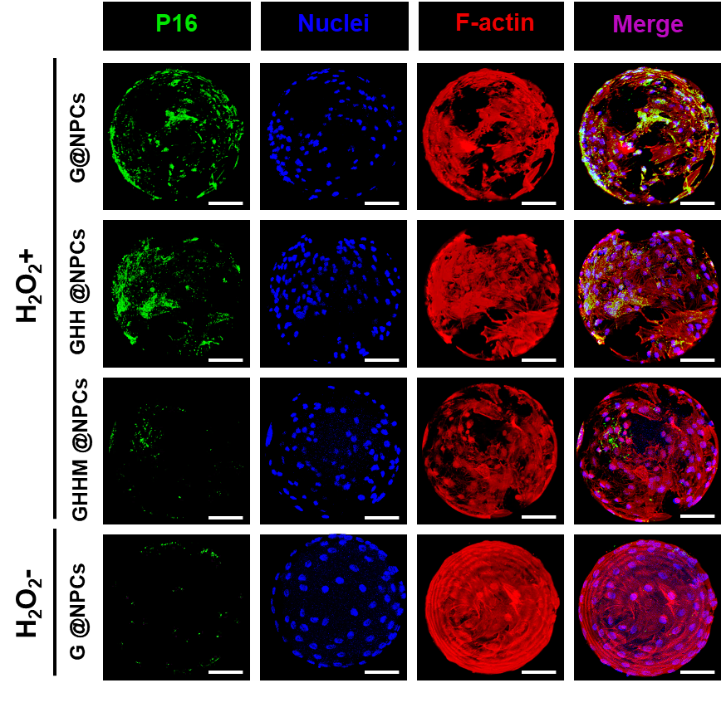


**Figure S4**. P21 immunofluorescence images after 200μM H_2_O_2_ treatment, scale bar = 50μm.

**Figure S5**. Quantification of ROS levels in each group of S-NPCs after 200μM H_2_O_2_ treatment.

**Figure S6**. Quantification of ROS levels in nucleus pulposus tissue in situ 7 days after injection in different groups.

**
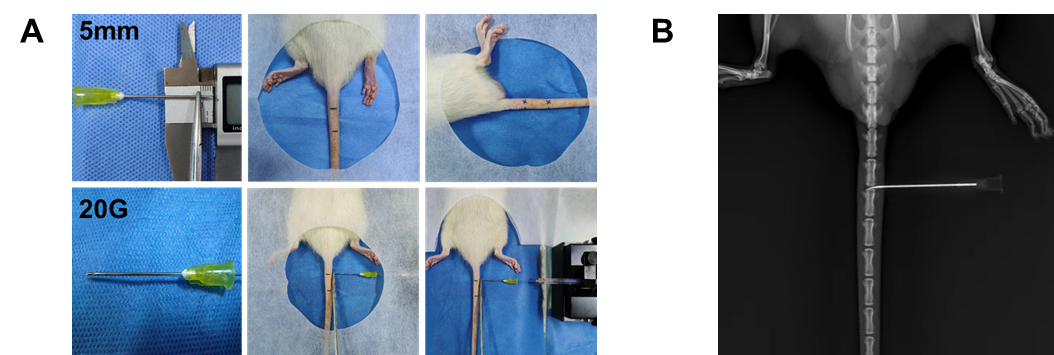
**

**Figure S7**. Establishment of a rat caudal degeneration model. A) Schematic diagram of animal experimental operations. B) Pictures of intraoperative x-ray localization.

 **Figure S8**. Calculation formula and diagram of DHI.

**Figure S9**. Quantification of the proportion of SA-β-Gal staining positive cells in each group of S-NPCs after 200μM H_2_O_2_ treatment.


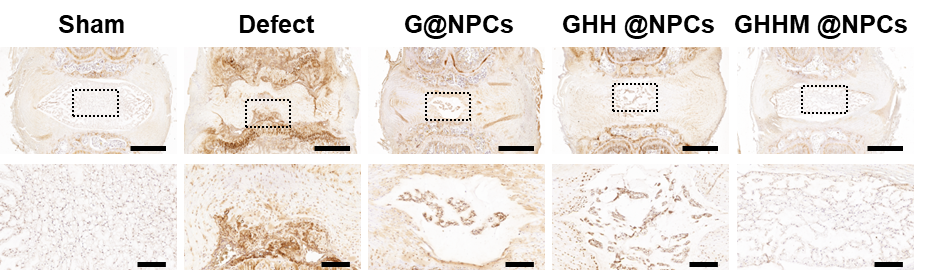


**Figure S10**. Immunohistochemical staining images of MMP13 at 4weeks, above scale bar = 1mm, below scale bar = 200μm.
